# A Comprehensive Review on Chemotaxonomic and Phytochemical Aspects of Homoisoflavonoids, as Rare Flavonoid Derivatives

**DOI:** 10.3390/ijms22052735

**Published:** 2021-03-08

**Authors:** Javad Mottaghipisheh, Hermann Stuppner

**Affiliations:** Institute of Pharmacy/Pharmacognosy, Center for Molecular Biosciences (CMBI), University of Innsbruck, Innrain 80–82, 6020 Innsbruck, Austria; Hermann.Stuppner@uibk.ac.at

**Keywords:** homoisoflavonoids, chemotaxonomy, Asparagaceae, Fabaceae

## Abstract

Homoisoflavonoids (3-benzylidene-4-chromanones) are considered as an infrequent flavonoid class, possessing multi-beneficial bioactivities. The present study gives an overview on phytochemical aspects of homoisoflavonoids, including utilized plant species, parts, extracts, and separation techniques. Overall, these compounds have mainly been isolated and identified from bulbs and rhizomes of the plants belonging to Asparagaceae and Fabaceae families, particularly the genera of *Ophiopogon*, *Dracaena*, *Scilla*, *Polygonatum*, and *Caesalpinia*.

## 1. Introduction

**Figure 1:**
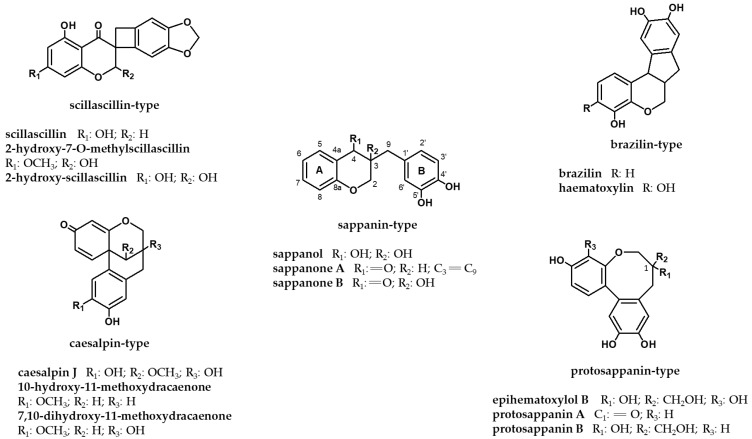
Homoisoflavonoid classification with some representatives.

Homoisoflavonoids (HIFs), a small, rare, and unique class of the flavonoids, are naturally occurring oxygen heterocyclic compounds possessing two aromatic rings, and an additional carbon between the B and C rings on the isoflavonoid skeleton (Figure 1) [1,2]. As demonstrated in Figure 1, the most updated classification of HIFs is categorized into five groups: sappanin, scillascillin, brazilin, caesalpin, and protosappanin types [3,4].

Biological studies performed with representatives of this compound class indicated potent antifungal (e.g., caesalpinianone, and 6-*O*-methylcaesalpinianone) [5], antioxidant (e.g., intricatinol and intricatin [6], 7-hydroxy-3-[(3,4-dihydroxyphenyl)methyl]-4*H*-chromen-4-one, 7,8-dihydroxy-3-[(4-hydroxyphenyl)methyl]-4*H*-chromen-4-one, and 7,8-dihydroxy-3-[(4-methoxyphenyl)methyl]-4*H*-chromen-4-one [7]), antiviral (e.g., 3-(4′-hydroxybenzyliden)chroman-4-one and 3-(4′-hydroxybenzyl) chroman-4-one) [8], antiproliferative (e.g., synthesized derivatives 3-(4′-hydroxybenzyliden)chroman-4-one and 3-(4′-hydroxybenzyl) chroman-4-one)) [9], anti-inflammatory (e.g., ophiopogonanone G, ophiopogoside A, and ophiopogoside B) [10], antihistaminic, antiallergic (e.g., synthesized 3-benzyl-chromone derivatives) [11], cytotoxicity (e.g., synthesized 3-benzyl-chromone derivatives) [8], antimutagenic (e.g., intricatin and intricatinol [12], 3-benzylidene-4-chromanones [13]), protein tyrosine kinase (PTK)-inhibiting (e.g., hematoxylin [14], (*E*)-3-(3,4-dihydroxybenzylidene)-7-methoxychroman-4-one, (*E*)-3-(3,4-dihydroxybenzylidene)-7-propoxychroman-4-one, (*E*)-3-(4-hydroxybenzylidene)-7-methoxychroman-4-one, and (*E*)-3-(4-hydroxybenzylidene)-chroman-4-one [15]), and antiangiogenic activities (e.g., cremastranone) [16].

The major aim of this paper is to gather the most updated information on chemosystematic and phytochemical features of HIFs in order to facilitate future scientific works. Contrary to the papers published [1,17,18] which deal with the phytochemistry and biological potencies of these compounds, the emphasis of this review is on separation techniques implemented for isolation and purification of different homoisoflavonoid (HIF) derivatives from diverse plant species in detail.

The keyword of “homoisoflavonoid” was applied to search the associated published data through databases covering PubMed, Web of Science, and SciFinder since 1980 (last search: 31 January 2021).

## 2. Separation Techniques Used for the Isolation and Purification of Homoisoflavonoids

Given that HIFs are mostly polar compounds, polar organic solvents have been used for the isolation and purification. Besides crystallization methods, the following chromatographic techniques were predominantly applied: semi-preparative and preparative column chromatography (CC) using reverse phase material as stationary phase and methanol (MeOH), water (H_2_O), and acetonitrile (MeCN) as mobile phase solvents; normal phase CC on silica gel using MeOH, chloroform (CHCl_3_), dichloromethane (CH_2_Cl_2_), acetone (Me_2_CO), etc. as eluent; Sephadex^®^ LH-20 (SLH) with MeOH, ethanol (EtOH), and CH_2_Cl_2_ as a solvent system; flash chromatography (FC) and preparative thin layer chromatography on normal (PTLC) and reverse phases (RP-PTLC). These methods are discussed in detail in the following sections.

## 3. Chemotaxonomy of Homoisoflavonoids

Based on the literature, HIFs have been isolated mainly from Asparagaceae, Fabaceae (syn. Leguminosae), and Portulacaceae families.

### 3.1. Homoisoflavonoids Isolated from Asparagaceae Family

The Asparagaceae family, belonging to the Asparagales order, comprises over 153 genera and 2500 species of flowering plants. This large family is distributed nearly all around the world [19]. 

Overall, HIFs have been isolated and identified from 23 genera and 49 species within to this family. Most of the identified compounds were isolated from *Ophiopogon japonicus*, *Polygonatum* spp. (mainly *P. odoratum*), *Scilla* spp., *Dracaena* spp., and *Bellevalia* spp. Among them, bulbs of *Ophiopogon japonicus*, determined to contain almost 60 HIFs, are deemed as the richest natural source of this compound class.

**Figure 2:**
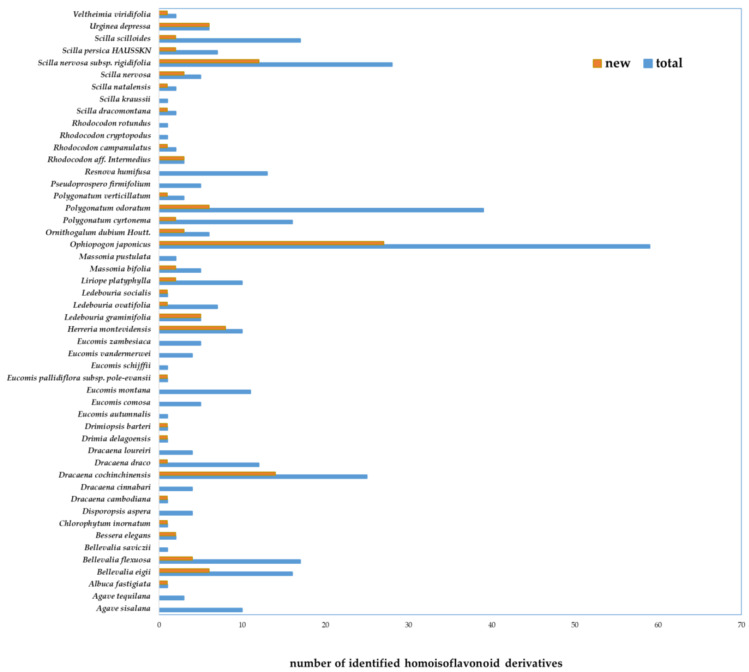
Abundance comparison of the novel homoisoflavonoids (identified for the first time) with total derivatives isolated from species of Asparagaceae family.

Details about single species, investigated plant parts and soluble fractions, applied chromatographic techniques, and names of the isolated compounds are listed in Table S1. Figure 2 exhibits the abundance variation of HIFs isolated from different species of Asparagaceae.

#### 3.1.1. *Ophiopogon japonicus*

The species of *Ophiopogon japonicus* is known as “Maidong” in China and has diverse folk medicinal applications such as fever treatment in consumptive ailments or general debility, dehydration of febrile disease, and dry mouth [20]. This species is considered as the major source of HIF compounds. Its tuberous roots are rich in these constituents, explicitly, ethyl acetate (EtOAc) and hydro-alcoholic soluble fractions. From the ether extract using CC on silica gel (eluent: benzene (Bz)), followed by recrystallization, methylophiopogonone A, methylophiopogonanone A, and a novel derivative, namely, 5-hydroxy-7,8-dimethoxy-6-methyl-3-(3′,4′-dihydroxybenzyl)chroman-4-one have been isolated. Ophiopogonanone A and ophiopogonone A were also isolated through silica gel CC using *n*-hexane (NHEX)–Me_2_CO (8:2) as mobile phase and recrystallization. Moreover, semi-preparative HPLC was applied to separate desmethylisoophiopogonone B, and new derivatives of 5,7,2′-trihydroxy-6-methyl-3-(3′,4′-methylenedioxybenzyl)chromone and 5,7,2’-trihydroxy-8-methyl-3-(3′,4′-methylenedioxybenzyl)chromone [21].

Sephadex^®^ LH–20 (SLH), as a gel filtration chromatographic technique, has been widely utilized for successful separation of phytochemicals according to their molecular sizes. This method is deemed as one of the most convenient, cheap, prompt, and efficient procedures for isolation and purification of flavonoids including HIFs and their derivatives [22]. Previously, five novel HIFs were isolated and identified from EtOH-EtOAc fractions of *O. japonicus*: ophiopogonanone C by recrystallization, ophiopogonanone D and ophiopogonone C with SLH (mobile phase: MeOH–H_2_O 4:1), ophiopogonanone E and ophiopogonanone F, along with known 5,7,2′-trihydroxy-6-methyl-3-(3′,4′-methylenedioxybenzyl) chromone and 2′-hydroxymethylophiopogonone A via PTLC with CHCl_3_–EtOAc (20:1) as mobile phase, and 6-aldehydoisoophiopogonone A by applying silica gel CC with NHEX–CHCl_3_ as eluting solvent system mobile phase [20].

Reverse-phase CC (RP-CC) on silica gel has been carried out to isolate four new HIF derivatives from the CHCl_3_-soluble partition of the tuber part; a homoisoflavanone homoisopogon A and one homoisoflavan homoisopogon C, and two homoisoflavans named homoisopogon B and D were consequently purified by eluting gradient mobile phase H_2_O–Me_2_CO (2:3 and 1:1), respectively [23]. The EtOAc-soluble fraction of the roots also contained ophiopogonone D and ophiopogonanone G, isolated as the new secondary metabolites. In this study, the extract was initially subjected to CC on silica gel by increasing the ratio of Me_2_CO in petroleum ether (PET) as mobile phase, then chromatographed with CC on polyamide, where H_2_O–MeOH (10:0 to 0:10) was applied as mobile phase, and, finally, the aforementioned compounds were purified by SLH (eluent: MeOH) and repeated HPLC via an isocratic mobile phase MeOH in H_2_O (7:3) [24].

In the study of Hoang Anh et al. [25], 13 HIF derivatives were isolated and characterized from the root EtOAc extract; among them, four compounds, 5,7-dihydroxy-8-methoxy-6-methyl-3-(2′-hydroxy-4′-methoxybenzyl)chroman-4-one, 7-hydroxy-5,8-dimethoxy-6-methyl-3-(2′-hydroxy-4′-methoxybenzyl)chroman-4-one, 5,7-dihydroxy-6,8-dimethyl-3-(4′-hydroxy-3′-methoxybenzyl)chroman-4-one, and 2,5,7-trihydroxy-6,8-dimethyl-3-(3′,4′-methylenedioxybenzyl)-chroman-4-one, were identified for the first time in nature, where flash chromatography (FC) (mobile phase: CHCl_3_–MeOH), PTLC (mobile phase: CHCl_3_–MeOH), and SLH (eluent: ethanol) were utilized for the isolation.

H_2_O–MeCN (0.1% formic acid (HCO_2_H)) has previously been implemented as the eluent system of HPLC to isolate three novel compounds, ophiopogonanone G (ratio: 25:75), ophiopogoside A, and ophiopogoside B (ratio: 20:80), from the root EtOAc-soluble partition [10]. Two new derivatives, ophiopogonone E and ophiopogonanone H, have been isolated and characterized for the first time from hydro-ethanolic (80%) soluble fractions of the tuberous root, by applying a gradient system H_2_O–MeOH (40:60 to 30:70) using a C18 column in semi-prep HPLC; whereas silica gel CC was primarily exploited to fractionate the extract eluting with CHCl_3_–MeOH (1:0 to 1:1) as mobile phase. Thereafter, SLH (eluent: MeOH), and lastly preparative HPLC with H_2_O–MeOH (40:60 to 30:70) as mobile phase were used to isolate both novel constituents. Moreover, 13 other identified HIFs have been separated through HPLC (mobile phase: H_2_O–MeOH), and SLH (eluent: MeOH) [26].

High-speed counter-current chromatography (HSCCC) has been employed to isolate three known derivatives comprising methylophiopogonanone A, 6-aldehydo-isoophiopogonone A, and 6-formyl-isoophiopogonanone A from *O. japonicus* methanolic extract; where a two-phase elution system of NHEX–EtOAc–MeOH–MeCN–H_2_O (1.8:1.0:1.0:1.2:1.0) was exploited [27]. Aside from the abovementioned fractions, diethyl ether (Et_2_O) of the tuber part was subjected to isolation of its major constituents; thereby, four novel HIFs, namely, methylophiopogonanone A and B using SLH (eluents: MeOH and EtOH), as well as methylophiopogonone A and B by applying SLH (eluent: MeOH) and CC on silica gel via mobile phase system CHCl_3_–Me_2_CO (95:5), were isolated [28].

4′-*O*-Demethylophiopogonanone E, a new natural product, along with eight previously described HIF derivatives, was isolated from a hydro-ethanolic (70%) extract of *O. japonicus* by first developing silica gel CC via CHCl_3_–MeOH (100:0 to 85:15), then SLH with CHCl_3_–MeOH (1:1) as eluent systems [29]. Likewise, silica gel CC applying the gradient system PET–EtOAc (50:1, 20:1, 10:1, 5:1, 2:1), and finally HSCCC (eluting solvent system: NHEX–EtOAc–MeOH–MeCN–H_2_O 3:2:3.5:1:0.5; 3:2:2.5:1:1.5) led to purification of four known HIFs [30].

#### 3.1.2. *Polygonatum* spp.

##### *Polygonatum* *odoratum*

*Polygonatum odoratum* is famed for therapeutic characteristics for upset stomachs, lung illnesses, palpitations, and diabetes. HIFs have been described as one of its main constituents. From the PET fractions of the rhizome, two novel homoisoflavanones, odoratumone A and B, have been isolated [31]. Silica gel CC (gradient solvent systems: PET–Me_2_CO (98:2 to 50:50) and PET–EtOAc (50:1 to 5:1)) of the EtOAc extract of *P. odoratum* rhizome, followed by SLH (eluent: CHCl_3_–MeOH 4:1), led to the isolation of the new *C*-methylated homoisoflavanone methylodoratumanone A. Moreover, four known methyl-homoisoflavanones, 3-(4′-hydroxy-benzyl)-5,7-dihydroxy-6,8-dimethyl-chroman-4-one, 3-(4′-hydroxy-benzyl)-5,7-dihydroxy-6-methyl-8-methoxy-chroman-4-one, and 3-(4′-hydroxy-benzyl)-5,7-dihydroxy-6-methyl-chroman-4-one, could be obtained as pure compounds [32].

Rafi and Vastano [33] separated 2,3-dihydro-3-[(15-hydroxyphenyl)methyl]-5,7-dihydroxy-6-methyl-8-methoxy-4H-1-benzopyran-4-one by means of semi-prep HPLC (isocratic mobile phase system: H_2_O–MeCN 60:40) from an EtOAc-soluble fraction of the roots. In another study, nine formerly identified HIFs were isolated from EtOAc extract of *P. odoratum* root using silica gel CC (mobile phase: CHCl_3_–MeOH–H_2_O and PET–EtOAc), SLH (eluent: CHCl_3_–MeOH; MeOH), and PTLC (mobile phase: CHCl_3_–MeOH) [34].

Diverse chromatographic techniques including SLH, PTLC, and HPLC were exploited to isolate the known compounds (*E*)-3-(3,4-dihydroxybenzylidene)-5,7-dihydroxy-6,8-dimethylchroman-4-one and (*E*)-3-(3,4-dihydroxybenzylidene)-5,7-dihydroxy-8-methoxy-6-methylchroman-4-one from a CHCl_3_ fraction of the rhizome [35], whereas (3*R*)-5,7-dihydroxyl-6-methyl-8-methoxyl-3-(4′-hydroxylbenzyl)-chroman-4-one, (3*R*)-5,7-dihydroxyl-6,8-dimethyl-3-(4′-hydroxylbenzyl)-chroman-4-one, (3*R*)-5,7-dihydroxyl-6-methyl-3-(4′-hydroxylbenzyl)-chroman-4-one, and polygonatone A–C were acquired from ethanolic soluble fractions of *P. odoratum* rhizomes [36].

Application of PTLC and silica gel CC (mobile phase: NHEX–EtOAc) led to the purification of 5,7-dihydroxy-6,8-dimethyl-3-(4′-hydroxybenzyl)chroman-4-one and 5,7-dihydroxy-6-methyl-8-methoxy-3-(4′-hydroxybenzyl)chroman-4-one from a *P. odoratum* EtOAc extract [37]. The rhizome EtOAc fraction of *P. odoratum* has been targeted by RP-HPLC by applying isocratic solvent system H_2_O–MeCN (40:60), and three known HIFs were subsequently isolated [38]. Separation of the CHCl_3_-soluble fraction of the rhizome by means of RP-CC and HPLC (solvent gradients of H_2_O–MeOH 40:60, 35:65, 30:70, and 30:70 to 0:100, respectively) resulted in the isolation of three previously characterized derivatives, namely, 3-(4′-hydroxybenzyl)-5,7-dihydroxy-6-methyl-8-methoxychroman-4-one, 3-(4′-hydroxybenzyl)-5,7-dihydroxy-6,8-dimethylchroman-4-one, and 3-(4′-methoxybenzyl)-5,7-dihydroxy-6-methyl-8-methoxychroman-4-one [39].

HSCCC (solvent system: PET–EtOAc–MeOH–H_2_O 2:3:3:2) and SLH (eluent: MeOH–MeCN 1:1) of an hydro-ethanolic (60%) extract of *P. odoratum* rhizome yielded three novel HIFs, (3*R*)-5,7-dihydroxy-8-methyl-3-(2′,4′-dihydroxybenzyl)-chroman-4-one, (3*R*)-5,7-dihydroxy-8-methyl-3-(4′-hydroxybenzyl)-chroman-4-one, and (3*R*)-5,7-dihydroxy-3-(2′-hydroxy-4′-methoxybenzyl)-chroman-4-one, along with eight known derivatives [40].

##### *Polygonatum* *cyrtonema*

(3*R*)-5,7-Dihydroxy-8-methyl-3-(2′-hydroxy-4′-methoxybenzyl)-chroman-4-one has been purified as a novel homoisoflavanone from an EtOAc extract of the *P. cyrtonema* rhizome by way of silica gel CC with the mobile phases PET–EtOAc and NHEX–Me_2_CO, followed by PTLC (eluent: NHEX–Me_2_CO 3:1) [41]. Wang et al. [42] reported the first isolation of polygonatone H from a rhizome’s PET-soluble fraction of *P. cyrtonema* via CC on silica gel and semi-prep HPLC. In this study, eight previously described derivatives from the abovementioned fraction, and six compounds from the EtOAc extract, were also isolated.

##### *Polygonatum* *verticillatum*

CHCl_3_ and EtOAc soluble fractions obtained from *P. verticillatum* were chromatographed on CC silica gel (mobile phase: Bz–EtOAc 10:0 to 0:10) and RP-18 (elution system: H_2_O–MeOH 70:30) to obtain 5,7-dihydroxy-3-(4-methoxybenzyl)-8-methyl chroman-4-one as a new natural product. CC on silica gel was also used to isolate two other known HIFs [43].

#### 3.1.3. *Scilla* spp.

To date, 16 HIFs have been separated and identified from the bulb part of *Scilla nervosa* subsp. *Rigidifolia*: 3-(4-hydroxybenzylidene)-5,7-dihydroxy-6-methoxychroman-4-one, 3-(4-methoxybenzylidene)-5,7-dihydroxy-6-methoxychroman-4-one, 3-(4-hydroxybenzylidene)-5,7-dihydroxychroman-4-one, 3-(4-hydroxybenzylidene)-5-hydroxy-7-methoxychroman-4-one, 3-(4-methoxybenzyl)-5,7-dimethoxychroman-4-one, 3-(4-hydroxy-3-methoxybenzyl)-5-hydroxy-7-methoxychroman-4-one, 3-(3-hydroxy-4-methoxybenzyl)-5,7-dihydroxychroman-4-one, 3-(4-hydroxy-3-methoxybenzyl)-5-hydroxy-6,7-dimethoxychroman-4-one, 3-(4-hydroxybenzyl)-5,7-dihydroxy-6-methoxychroman-4-one, 3-(3,4-dimethoxybenzyl)-5,7-dihydroxychroman-4-one, 3-(4-methoxybenzyl)-6-hydroxy-5,7-dimethoxychroman-4-one, 3-(4-hydroxybenzyl)-5,6,7-trimethoxychroman-4-one, 3-(4-methoxybenzyl)-8-hydroxy-5,7-dimethoxychroman-4-one, 3-(4′-methoxybenzyl)-6,7-dihydroxy-5-methoxychroman-4-one, 3-(4′-methoxybenzyl)-5,6,7-trimethoxychroman-4-one, and 3-(4′-methoxybenzyl)-7-hydroxy-5,6-dimethoxychroman-4-one. Isolation, separation, and purification of the single constituents were carried out by means of silica gel and SLH CC and PTLC, as well as FC [44,45,46,47,48,49,50,51].

#### 3.1.4. *Bellevalia* spp.

Sixteen HIFs were isolated from a CHCl_3_ extract of *Bellevalia eigii* bulbs. Seven were new constituents, 7-*O*-methylpunctatin, 7-*O*-methyl-3′-hydroxy-3,9-dihydropunctatin, 6-hydroxy-7-*O*-methyl-3,9-dihydropunctatin, 7,4′-di-*O*-methyl-3′-hydroxy-3,9-dihydropunctatin, 7-*O*-methyl-3′-hydroxypunctatin, 5-hydroxy-7,8-dimethoxychroman-4-one, and 7-*O*-methyl-8-demethoxy-3-hydroxy-3,9-dihydropunctatin, along with nine known derivatives. Partitioning chromatography followed by silica gel CC (gradient solvent system of NHEX, CHCl_3_, and MeOH) and C18 semi-prep HPLC (H_2_O-MeOH as mobile phase) led to the isolation of the pure compounds [52].

HPLC has been applied to isolate major constituents of *Bellevalia flexuosa.* A CHCl_3_ fraction obtained from the bulb yielded 7,4′-*O*-dimethyl-8-demethoxy-3,3′-dihydroxy-3,9-dihydropunctatin (mobile phase: H_2_O–MeCN 60:40 to 50:50), 7-*O*-methyl-8-demethoxy-3′-hydroxy-3,9-dihydropunctatin (mobile phase: H_2_O–MeCN 60:40 to 40:60), 6-hydroxy-8-demethoxy-4′-*O*-methyl-3,9-dihydropunctatin (mobile phase: H_2_O–MeCN 70:30 to 60:40), and 7-*O*-methyl-3-hyroxy-3,9-dihydropunctatin (mobile phase: H_2_O–MeCN 72:28) [53]. The known HIF dracol (identified from *Dracaena draco* for the first time, which will be mentioned in the next section) was also isolated from CH_2_Cl_2_ root extract of *Bellevalia saviczii* by exploitation of RP medium-pressure liquid chromatography (MPLC) with a gradient solvent system of H_2_O–MeOH from 80:20 to 0:100 [54].

#### 3.1.5. *Dracaena* spp.

Cambodianol was isolated for the first time from an EtOAc extract of *Dracaena cambodiana* stems. Silica gel CC (mobile phase: CHCl_3_–MeOH 50:1 to 30:1) and SLH (eluent: EtOH) were utilized as chromatographic tools [55].

The stems of *D. cinnabari* mainly include reddish resin. Four previously identified HIFs have been isolated from the CHCl_3_-soluble fractions of the resin by utilizing silica gel CC (mobile phase: NHEX–EtOAc) and HPLC (mobile phase: H_2_O–MeOH) [56]. The red resin of *Dracaena cochinchinensis* is commonly known as Chinese dragon’s blood (Longxuejie in Chinese). A variety of medicinal consumptions have been documented in Chinese folk medicine of its resin, specifically in promoting blood circulation and removing blood stasis [57]. Four meta-homoisoflavans consisting of three novel compounds, (7*R*,12b*R*)-7,10-dihydroxy-4,11-dimethoxydracaenone, (7*S*,12b*S*)-11-hydroxy-1,10-dimethoxydracaenone, and (7*S*,12b*S*)-10,11-dihydroxy-1-methoxydracaenone, have been separated from the resin EtOAc extract of this species by applying SLH (eluent: MeOH) and HPLC (mobile phase: H_2_O–MeCN 65:35). The use of MeOH as eluting solvent in SLH and HPLC (mobile phase: H_2_O–MeOH 42:58) also led to the isolation of a new homoisoflavanone and one novel homoisoflavan, namely, (3*S*)-3,7,4′-trihydroxy-5-methoxyhomoisoflavanonol and (3*R*)-7,4′-dihydroxy-6-methoxyhomoisoflavane, respectively [58].

In a similar investigation, novel constituents dracaeconolide A and B, together with four known derivatives, were isolated from the EtOAc fraction of *D. cochinchinensis* resin, using C18 RP silica gel as stationary phase and H_2_O–MeOH (6:4 to 0:1) and H_2_O–MeOH (1:1 to 0:1) as mobile phases [59]. The MeOH-soluble part of its reddish resin has further been subjected to SLH (eluent: MeOH) and HPLC (mobile phase: H_2_O–MeCN 56:44 to 53:47; 50:50; H_2_O–MeOH 35:65 to 33:67), and, subsequently, six new HIF dimers with chalcones, namely, biflavocochin A, B, D–G were isolated [60]. Likewise, by means of silica gel and SLH CC, Zheng et al. [61] isolated a new meta-homoisoflavan, namely, 10,11-dihydroxydracaenone C, with known homoisoflavone (7,4′-dihydrohomoisoflavanone) and homoisoflavan (7,4′-homoisoflavane) derivatives from EtOAc extract of *D. cochinchinensis* stem.

Dracol (syn. (3*R*)-2,3-dihydro-3,5-dihydroxy-7-methoxy-3-[(4-methoxyphenyl)-methyl]-8-methyl-4H-[1]benzopyran-4-one) was isolated for the first time from an ethanolic extract of the leaves of *D. draco* by developing SLH CC (eluent: NHEX–CH_2_Cl_2_–MeOH 2:1:1) and PTLC (mobile phase: NHEX–EtOAc 85:15) [62]. Ten previously identified HIFs were isolated from the Me_2_CO soluble part of the resin of *D. draco* [63]. Furthermore, from the *D. loureiri* stems, four known derivatives were isolated using silica gel CC and PTLC as separation tools [64].

#### 3.1.6. *Eucomis* spp.

The novel compound 8-methoxy-5,6,7-trihydroxy-3-(4′-hydroxybenzylidene)-4-chromanone was purified from *Eucomis pallidiflora* subsp. *pole*-*evansii*. In this study, the bulbs were extracted with solvents of different polarities, and the CH_2_Cl_2_ fraction was subjected to silica gel CC using CH_2_Cl_2_–MeOH (98:2) to isolate the mentioned constituent [65]. Moreover, some identified HIFs were isolated from the bulb parts of other *Eucomis* species. The following extracts were investigated: *E. autumnalis* (1-butanol) [66], *E. comosa* (EtOAc) [65], *E. montana* (CH_2_Cl_2_, EtOAc, and MeOH) [67], *E. schijffii* (CH_2_Cl_2_) [65], *E. vandermerwei* (CH_2_Cl_2_ and MeOH) [68], and *E. zambesiaca* (MeOH) [68].

#### 3.1.7. *Ledebouria* spp.

Chromatographic separation on SLH, then PTLC (CHCl_3_–MeOH 95:5), has led to the isolation of five novel HIFs, including 5-hydroxy-7-methoxy-3-(4′-hydroxybenzyl)-4-chromanone, 5-hydroxy-6,7-dimethoxy-3-(4′-hydroxybenzyl)-4-chromanone, 5,7,8-trimethoxy-3-(4′-hydroxybenzyl)-4-chromanone, 5-hydroxy-3′,4′,7-trimethoxyspiro{2H-1-benzopyran-7′-bicyclo[4.2.0]octa[1,3,5]-trien}-4-one, and 5,7-dihydroxy-3′,4′-dimethoxyspiro{2H-1-benzopyran-7′-bicyclo[4.2.0]octa[1,3,5]-trien}-4-one, from the CH_2_Cl_2_–MeOH fraction of *Ledebouria graminifolia* bulb [69].

A new derivative ovatifolionone (syn. (*E*)-3-(3′,4′-dihydroxybenzylidene)-5,7-dihydroxychroman-4-one) has been isolated and characterized from bulb EtOAc extract of *L. ovatifolia*, however, five previously identified HIFs have also been isolated from this plant [67]. In the same study, socialinone (syn. (*R*)-2′,5-dihydroxy-3′,4′,7-trimethoxyspiro{2H-1-benzopyran-3-(4H)-9-bicyclo[4.2.0]octa[1,3,5]triene}-4-one) was isolated as a novel constituent from the CH_2_Cl_2_ fraction of *L. socialis* bulbs, by applying silica gel CC (NHEX–CH_2_Cl_2_; CH_2_Cl_2_–MeOH), and finally PTLC (EtOAc–CH_2_Cl_2_ 10:90) [70].

#### 3.1.8. *Massonia* spp.

The ethanolic extract of *Massonia bifolia* bulb afforded two novel HIFs, (*E*)-3-benzylidene-(3′,4′-dihydroxy)-5-hydroxy-7-methoxy-4-chromanone and (*E*)-3-(3′,4′-dihydroxybenzylidene)-5-hydroxy-7-methoxy-4-chromanone, along with three known derivatives. Silica gel (CH_2_Cl_2_–EtOAc) and SLH CC (CH_2_Cl_2_–MeOH 1:1) were carried out as separating processes [69]. Similarly, two formerly identified HIFs could be isolated from the CH_2_Cl_2_ extract of *M. pustulata* bulb by applying silica gel and SLH CC [71].

#### 3.1.9. *Agave* spp.

The leaves of *Agave sisalana* were extracted with MeOH and partitioned between H_2_O and EtOAc. The organic phase was then subjected to silica gel CC by utilizing a mixture of NHEX–EtOAc–Me_2_CO–MeOH. Subfractions were subjected to HPLC separation (mobile phase: NHEX–EtOAc; NHEX–EtOAc–Me_2_CO) to obtain seven known HIFs [72]. Furthermore, Morales-Serna et al. [73] isolated three known compounds from EtOAc and Me_2_CO extracts of *A. tequilana* fruit by employing silica gel CC (eluent: NHEX–EtOAc 9:1 to 1:9) and FC (mobile phase: NHEX–EtOAc 8:2).

#### 3.1.10. *Rhodocodon* spp.

Numerous HIFs have been isolated from this genus. To date, three novel compounds named (*E*)-5,6,7-trihydroxy-3-(3′-hydroxy-4′-methoxybenzylidene)-4-chromanone, (*E*)-5,7-dihydroxy-3-(3′-hydroxy-4′-methoxybenzylidene)-4-chromanone, and (3*S*)-5,6-dihydroxy-7-methoxy-3-(3′-hydroxy-4′-methoxybenzyl)-4-chromanone have been isolated from a CH_2_Cl_2_ extract of *Rhodocodon aff. Intermedius* bulb by applying FC (NHEX, NHEX–CH_2_Cl_2_, MeOH) and silica gel CC (MeOH–NHEX–CH_2_Cl_2_ 1:4:5) [74]. One novel derivative, (3*S*)-5,7-dihydroxy-3*S*-(3′-hydroxy-4′-methoxybenzyl)-4-chromanone, was attained from an ethanolic fraction of *R. campanulatus* bulbs using SLH (CH_2_Cl_2_–MeOH 1:1) and silica gel CC [74]. Moreover, 2,5,7-dihydroxy-3-(3-hydroxy-4-methoxybenzyl) chroman-4-one, (3*S*)-5,7-dihydroxy-(3′-hydroxy-4′-methoxybenzyl)-4-chromanone, and (3*S*)-5,7-dihydroxy-(4′-hydroxy-3′-methoxybenzyl)-4-chromanone were isolated from bulb ethanolic extracts of *R. campanulatus* [75], *R. cryptopodus*, and *R. rotundus*, respectively [76].

#### 3.1.11. Miscellaneous Species from Asparagaceae Family

*Albuca fastigiate* is an endemic plant to South Africa, well known for its potency in the treatment of idliso (an illness caused by severe food poisoning) [77]. Utilizing silica gel CC (CH_2_Cl_2_–MeOH 99:1), the CH_2_Cl_2_-soluble fraction of the bulbs was separated and a previously undescribed HIF, 3,5-dihydroxy-7-methoxy-3-(4′-hydroxy-3′-methoxybenzyl)-4-chromanone, could be identified [78]. A methanolic bulb extract of *Bessera elegans* turned out to contain novel HIFs, including (3*R*)-5,7-dihydroxy-6-methyl-3-(3′-hydroxy-4′-methoxybenzyl)chroman-4-one and a rare derivative containing a scillascillin-type skeleton, (3*R*)-5,7,3′-trihydroxy-4′-methoxy-6-methylspiro[2H-1-benzopyran-7′-bicyclo[4.2.0]octa[1,3,5]-trien]-4-one. Silica gel (eluting system: CHCl_3_–MeOH–H_2_O 100:10:1) and RP CC (H_2_O–MeOH 1:2) were employed as separation instruments [79].

In another phytochemical study, an NHEX-soluble fraction extracted from *Chlorophytum inornatum* root material was chromatographed via SLH and RP-PTLC (mobile phase: H_2_O–MeOH 15:85), and 3-(4′-methoxybenzyl)-7,8-methylenedioxy-chroman-4-one was consequently isolated as a novel HIF derivative [80]. Three rare methyl-homoisoflavanones, 3-(4′-hydroxy-benzyl)-5,7-dihydroxy-6-methyl-8-methoxy-chroman-4-one, 3-(4′-hydroxy-benzyl)-5,7-dihydroxy-6-methylchroman-4-one, and 3-(4′-hydroxy-benzyl)-5,7-dihydroxy-6,8-dimethyl-chroman-4-one, along with the known compound disporopsin, have been purified from the EtOAc extract of *Disporopsis aspera* rhizome, utilizing silica gel CC with the solvent system NHEX–EtOAc (1:0, 9:1, 8:2, 7:3, 6:4, 5:5) and PTLC (eluent: NHEX–EtOAc) [81]. The CH_2_Cl_2_-soluble partition obtained from bulbs of *Drimia delagoensis* has been separated by means of silica gel CC using EtOAc–CH_3_Cl (2:10) as mobile phase; 5,7-dihydroxy-(4-hydroxy-3-methoxybenzyl)chroman-4-one was isolated and identified as a new HIF [82].

A novel tetrahydroxy homoisoflavanone, 5,6,7-trihydroxy-3-(4-hydroxybenzyl)chroman-4-one, has also been isolated from MeOH–CHCl_3_ soluble fractions of leaves and bulbs of *Drimiopsis barteri;* silica gel CC (mobile phase: PET–CHCl_3_ 3:7) and PTLC (eluent: CHCl_3_–PET–MeOH 7:2:0.5) were applied as the chromatographic tools [83].

*Herreria montevidensis* root material has been extracted with EtOAc–MeOH (1:1) and the corresponding fraction partitioned between CHCl_3_ and H_2_O. The H_2_O phase was then lyophilized and the resulting powder extracted with MeOH. Six novel homoisoflavans, namely, (3*R*)-7-methoxy-3-(4-hydroxybenzyl)chroman, (3*R*)-7-hydroxy-5-methoxy-6-methyl-3-(4-hydroxybenzyl)chroman, (3*R*)-7-hydroxy-5-methoxy-6-methyl-3-(4-hydroxybenzyl)chroman, and (3*R*)-7-methoxy-3-(4-hydroxybenzyl)chroman, along with two known homoisoflavans, have been isolated and characterized from the CHCl_3_-soluble fractions by applying HPLC (mobile phase: H_2_O–MeOH 5:5, 3:7). Moreover, two novel homoisoflavans, 7-hydroxy-8-methoxy-3-(4-hydroxybenzyl)-3-chromen and 7-hydroxy-8-methoxy-3-(4-hydroxybenzyl)-3-chromen, could be isolated from the methanolic extract [84].

Two previously undescribed HIFs named (-)-liriopein A and B have been isolated from a hydro-ethanolic root extract of *Liriope platyphylla*; silica gel CC (mobile phase: CH_2_Cl_2_–MeOH 98:2), SLH (eluent: CH_2_Cl_2_–EtOAc–MeOH 1:1:6), and HPLC (mobile phase: H_2_O–MeOH 35:65) were used for the isolation of (-)-liriopein A, whereas silica gel CC with the mobile phase CH_2_Cl_2_–MeOH (96.5:3.5) and RP-CC (mobile phase: H_2_O–MeOH 25:75) were employed in the case of (-)-liriopein B. Nevertheless, in this study, six known derivatives were also isolated and identified [85]. The same research group described two previously explored HIFs from an EtOAc extract, obtained from the *Liriope platyphylla* aerial part [86].

The ethanolic-soluble fraction of *Ornithogalum dubium* Houtt. bulbs has been investigated for the presence of major constituents; three novel HIFs, namely, (3*R*)-3,5-dihydroxy-7-methoxy-3-(4′-hydroxybenzyl)-4-chromanone, (3*R*)-3-hydroxy-7-methoxy-3-(4-hydroxybenzyl)-4-chromanone 5-*O*-β-D-glucopyranoside, and (3*R*)-3-hydroxy-7-methoxy-3-(4-hydroxybenzyl)-5-*O*-β-D-glucopyranosyl-(1→6)-β-D-glucopyranoside-4-chromanone, along with three known derivatives, were isolated by means of silica gel CC (mobile phase: CH_2_Cl_2_) and SLH (eluent: MeOH–CH_2_Cl_2_) [87]. In another study, silica gel CC eluting with CH_2_Cl_2_–EtOAc led to the isolation of five known HIFs present in the CH_2_Cl_2_-soluble partition of bulbs of *Pseudoprospero firmifolium* [88].

Whole *Urginea depressa* parts have been extracted using different solvents and, finally, CH_2_Cl_2_ and NHEX fractions were exploited for the isolation of their predominant components. Six undescribed natural products have been isolated and identified as HIF derivatives: urgineanin A, D, and F by means of HPLC (mobile phases: H_2_O–MeCN 10:3.5 and H_2_O–MeCN 10:4) from the CH_2_Cl_2_ fraction; urgineanin B, C, and E via HPLC (mobile phases: H_2_O–MeCN 10:4 and H_2_O–MeCN 10:3.5) from the NHEX extract [89]. R(-)-3-(4-hydroxybenzyl)-5-hydroxy-6,7,8-trimethoxychroman-4-one was achieved by semi-prep HPLC eluting with NHEX–Et_2_O (8:2 to 0:10) from the bulb PET-soluble fraction of *Veltheimia viridifolia*. Furthermore, muscomin was isolated as a known derivative by application of CC on LiChroprep^®^ Si 60 from the Et_2_O fraction by employing the eluting solvent system CHCl_3_–MeOH (95:5) [90].

### 3.2. Homoisoflavonoids Isolated from Fabaceae Family

**Figure 3:**
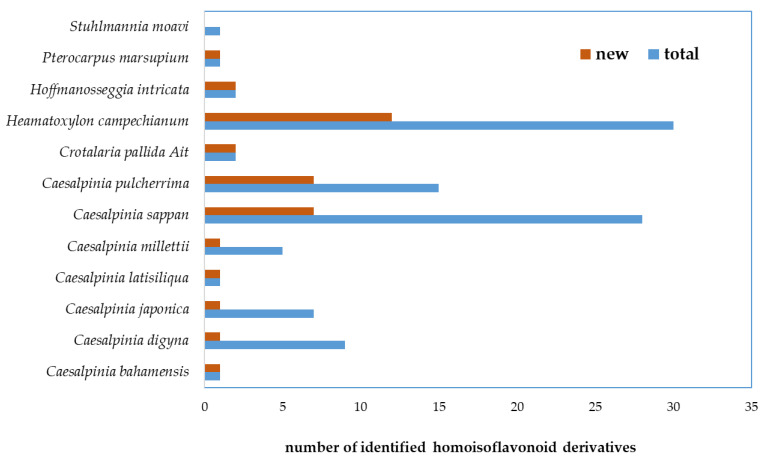
Abundance comparison of the novel homoisoflavonoids (identified for the first time) with total derivatives isolated from species of Fabaceae family.

A total of 39 novel and 57 previously identified HIFs have been isolated from 13 species belonging to the Fabaceae (syn. Leguminosae) family. Most of the isolated HIFs belong to *Heamatoxylon campechianum* and *Caesalpinia* spp. (Table S2, Figure 3).

#### 3.2.1. *Caesalpinia* spp.

Twenty-one new HIFs have been isolated from six *Caesalpinia* species. *C. sappan* and *C. pulcherrima* have been reported to be the richest species of this genus, each possessing eight novel derivatives among 28 and 15 identified HIFs, respectively.

##### *Caesalpinia* *bahamensis*

This species is traditionally consumed in Cuban folk medicine for the treatment of diabetes mellitus, hepatic problems, and peptic ulcers. Recently, Felipe González et al. [91] extracted its roots; liquid–liquid partitioning afforded a methanolic fraction which was subjected to a fractionation procedure. A novel HIF, metasappanin (syn. 3-(2-hydroxy-4-methoxybenzyl)chromane-4,7-diol) was isolated by applying FC (gradient solvent system of CH_2_Cl_2_–EtOAc) and HPLC (mobile phase H_2_O (0.1% HCO_2_H) and MeCN (0.1% HCO_2_H)).

##### *Caesalpinia* *bonduc*

Two novel HIFs, named caesalpinianone and 6-*O*-methylcaesalpinianone, have previously been isolated from the EtOH-soluble fraction of *Caesalpinia bonduc* bark; CC on silica gel using NHEX–CHCl_3_ and CHCl_3_–MeOH as eluent solvents, and finally PTLC (mobile phase: CH_2_Cl_2_–MeOH 75:25), were applied to isolate those compounds [5].

##### *Caesalpinia* *digyna*

A MeOH-soluble fraction of *Caesalpinia digyna* has been developed for isolation of its predominant constituents. The new HIF, isointricatinol (syn. (*Z*)-7,8-dihydroxy-3-(4′-methoxybenzyl)chroman-4-one), was isolated by using silica gel CC (gradient solvent system: NHEX–EtOAc 65:35 to 60:40). In addition, eight known derivatives were isolated and identified by means of CC on silica gel and SLH, as well as HPLC [92].

##### *Caesalpinia* *japonica*

In a study carried out by Namikoshi et al. [93], a CH_2_Cl_2_-soluble fraction of *Caesalpinia japonica* heartwood was chosen for preparative phytochemical analysis. Application of PTLC (eluent: CHCl_3_–EtOAc 9:1) led to the isolation of the undescribed HIF 3′-deoxy-4-*O*-methylsappanol (syn. 3,7-dihydroxy-3-(4-hydroxybenzyl)-4-methoxychroman) and the known derivative 4-*O*-methylepisappanol. Five more known HIFs were further isolated by using CC on silica gel and PTLC.

A novel homoisoflavanone glycoside named (3*S*)-dihydrobonducellin 8-*O*-β-D-glucopyranoside was isolated from an EtOAc extract of the twigs of *Caesalpinia latisiliqua* through an isocratic solvent system of MeCN–MeOH (35:65) as mobile phase on semi-prep HPLC [94]. Furthermore, five HIFs, including one new compound, 8-methoxyisobonducellin, could be obtained from a Me_2_CO extract derived from *Caesalpinia millettii* stems using SLH and MeOH as eluent [95].

##### *Caesalpinia* *sappan*

*Caesalpinia sappan* is famed for its heartwood “sappan lignum” in Southeastern Asia. This plant part is described as a red dyestuff, possessing diverse folk medicinal applications such as improving blood circulation and anti-allergic, anti-influenza, and neuroprotective activities [96,97,98,99]. Beside the diverse phytoconstituents identified from this plant, its heartwood can be considered as one of the major natural sources of HIF compounds. From an EtOAc-soluble fraction, sappanone A and B and 3-deoxysappanone B as homoisoflavanones, along with 3′-deoxy-4-*O*-methylsappanol, sappanol, 7,3′,4′-trihydroxy-3-benzyl-2H-chromene, episappanol, 4-(7-hydroxy-2,2-dimethyl-9βH-1,3,5-trioxa-cyclopenta[α]naphthalene-3-lymethyl)-benzene-1,2-diol, 4-*O*-methylepisappanol, and 4-*O*-methylsappanol, have been isolated by means of silica gel CC (mobile phase: CHCl3-MeOH), SLH (eluent: MeOH), and RP CC (mobile phases: H_2_O–MeOH and H_2_O–MeCN) [100].

In a similar experiment, HPLC separation (mobile phases: 30:70 and 40:60 of H_2_O–MeOH) of an EtOAc extract of the heartwood was conducted for the isolation of caesalpiniaphenol A and B [101]. HSCCC of an EtOAc-soluble partition of *C. sappan* heartwood applying CHCl_3_–MeOH–H_2_O (4:3:2) as mobile phase has also led to the separation of four previously described HIFs [102]. A novel homoisoflavan, namely, 7,3′,4′-trihydroxy-3-benzyl-2H-chromene, and three known derivatives, have been isolated and identified from the aforementioned plant extract, where CC on silica gel (mobile phase: CHCl_3_–MeOH 95:5; CHCl_3_–Me_2_CO 95:5, 92:8, 88:12), and SLH CC (eluent: H_2_O–MeOH 30:70) were implemented. In this study, another novel compound, 3′,4-di-*O*-methylepisappanol (syn. (3*R*,4*R*)-3,7-dihydroxy-3-(3′-methoxy-4′-hydroxybenzyl)-4-methoxychroman), was further isolated by applying silica gel and SLH CC, and lastly HPLC (mobile phase: H_2_O–MeOH 50:50) [103].

Moreover, a methanolic extract of the heartwood part has been chromatographed on SLH (eluent: MeOH) and separated by PTLC (mobile phase: CHCl_3_–Me_2_CO 2:1) to gain 7-hydroxy-3-(4′-hydroxybenzylidene-chroman-4-one, 3,7-dihydroxy-3-(4′-hydroxybenzyl)-chroman-4-one, and 3,4,7-trihydroxy-3-(4′-hydroxybenzyl)-chroman as new secondary metabolites, along with two known derivatives [104]. The previously discovered HIFs 4-*O*-methylsappanol, protosappanin A, brazilin, and caeasalpin J have also been isolated from *C. sappan* heartwood [105]. Additionally, centrifugal partition chromatography by employing the solvent system EtOAc–MeCN–H_2_O (1:1:2) as the mobile phase solvent system has been applied for the isolation of sappanol and brazilin from this species [106].

##### *Caesalpinia* *pulcherrima*

Aerial parts of *Caesalpinia pulcherrima* can be considered as the richest natural source for bonducellin and its derivatives. The aerial parts have been extracted by diverse solvents; after solvent–solvent partitioning, the CHCl_3_–MeOH soluble fraction was subjected to PTLC using different ratios of NHEX–EtOAc (10:3, 9:1, 10:4, 10:1.5) as mobile phase. Four novel HIFs, (3*E*)-3-(1,3-benzodioxol-5-ylmethylene)-2,3-dihydro-7-hydroxy-4H-1-benzopyran-4-one, (3*E*)-3-(1,3-benzodioxol-5-ylmethylene)-2,3-dihydro-7-methoxy-4H-1-benzopyran-4-one, (3*E*)-2,3-dihydro-7-hydroxy-3-[(3-hydroxy-4-methoxyphenyl)-methylene]-4H-1-benzopyran-4-one, and (3*E*)-2,3-dihydro-3-[(3,4-dimethoxyphenyl)methylene]-7-methoxy-4H-1-benzopyran-4-one, as well as five known derivatives, were isolated [107]. (*E*)-7-Methoxy-3-(4′-methoxybenzylidene)chroman-4-one and (*E*)-7-hydroxy-3-(3′,4′,5′-trimethoxybenzylidene)chroman-4-one were obtained from NHEX- and Me_2_CO-soluble partitions of *C. pulcherrima* whole parts, respectively; CC on silica (mobile phase: NHEX–EtOAc 95:5 and 60:40) was used as the separation tool [108].

CC on silica gel using NHEX–EtOAc (60:40, 65:35) as the solvent system has led to the isolation of isobonducellin and bonducellin, as novel and known HIFs, respectively, from a Me_2_CO fraction of aerial parts of *C. pulcherrima* [109]. Both compounds were also isolated from the same plant extract in other studies [110,111]. Bonducellin and 8-methoxybonducellin have been isolated from a CHCl_3_-soluble fraction of the stems using CC on silica gel with CHCl_3_–EtOAc as a solvent system, recrystallization, and PTLC (eluent: CHCl_3_–EtOAc 3:1) [112]. Moreover, five previously described HIFs have been isolated from a 1-butanol fraction of *C. pulcherrima* (cork tissue); CC on silica gel using NHEX–Me_2_CO (5:1 to 1:1) as a gradient solvent system, HPLC (mobile phase: H_2_O–MeCN 63:37), and PTLC (eluent: CHCl_3_–Me_2_CO 12:1) were applied [113].

##### *Caesalpinia* *latisiliqua*

The novel secondary metabolite (3*S*)-dihydrobonducellin-8-*O*-β-D-glucopyranoside has been purified as a homoisoflavanone glycoside from twigs of *C. latisiliqua*, whilst an EtOAc-soluble fraction was developed for silica gel (mobile phase: CHCl_3_–MeOH) and RP18 CC (H_2_O–Me_2_CO 1:1) as well as HPLC (H_2_O–MeCN 65:35) [94].

##### *Caesalpinia* *millettii*

8-Methoxyisobonducellin has been isolated and characterized for the first time from the Me_2_CO fraction of *C. millettii* stems; SLH CC (eluent: MeOH) was used as the final separation step. Moreover, four known HIFs consisting of 8-methoxybonducellin, eucomin, bonducellin, and intricatinol were isolated by means of CC on silica gel and SLH [95].

#### 3.2.2. *Crotalaria pallida* Ait

Two novel derivatives named cropalliflavone A and B have been isolated from a CH_2_Cl_2_-soluble partition of *C. pallida* Ait seeds; SLH CC (eluent: CH_2_Cl_2_–MeOH 2:1) and HPLC (mobile phase: H_2_O–MeOH 42:58) were employed as the chromatographic tools [114].

#### 3.2.3. *Heamatoxylon campechianum*

To date, a number of new HIFs have been isolated and identified from EtOAc- and CH_2_Cl_2_-soluble fractions of stems and heartwood of *Heamatoxylon campechianum*. HPLC with H_2_O–MeCN (90:10 to 65:35 and to 70:30) as mobile phase and SLH CC (eluent: MeOH) led to the isolation of hematoxylol, hematoxylone, isohematoxylin, epihematoxylol, 4-*O*-methylepihematoxylol, hematoxylene, hematoxin, 4-*O*-methylhematoxylol, and epihematoxin as novel HIFs. From the CH_2_Cl_2_ fraction, an undescribed derivative sappanene could be isolated using SLH CC (eluent: CHCl_3_–MeOH 1:1) as a final chromatographic step. Moreover, seven known HIFs were isolated from the extract by applying CC on silica gel (mobile phase solvent system: CHCl_3_–MeOH and Me_2_CO–PET) [115].

In the course of a newer study, the methanolic extract of the heartwood of *H. campechianum* has been subjected to silica gel (mobile phases: CHCl_3_-MeOH, CH_2_Cl_2_–Me_2_CO), SLH (eluent: CHCl_3_–MeOH 1:1), and RP-18 CC (mobile phase: H_2_O–MeOH 90:10 to 50:50), followed by an HPLC separation (mobile phase: H_2_O–MeCN 90:10 to 70:30). Epihematoxylol B, 1′-*O*-methylhematoxylol B, 1′-*O*-methylepihematoxylol B, hematoxylol A, 4-*O*-methylhematoxylol, and hematoxin could be obtained as pure compounds [116].

#### 3.2.4. *Hoffmanosseggia intricata*

Two HIFs, intricatin (syn. 7,4′-dimethoxy-8-hydroxyhomoisoflavone) and intricatinol (syn. 4′-methoxy-7,8-dihydroxyhomoisoflavone) have been isolated and characterized for the first time from a CHCl_3_-soluble fraction of *Hoffmanosseggia intricate* roots. Purification was accomplished by initial CC on silica gel using a gradient mobile phase system of CH_2_Cl_2_–MeOH (95:5 to 98:2), then a recrystallization method [12].

#### 3.2.5. *Pterocarpus marsupium*

Heartwood parts of *Pterocarpus marsupium* have been extracted and subjected to solvent–solvent partitioning. The Et_2_O-soluble fraction was chromatographed on silica gel by successively utilizing mobile phases of Bz and Bz–EtOAc (7:3), resulting in the isolation of the novel HIF pteromarsupone (syn. 6-hydroxy-7-*O*-methyl-3-(3-hydroxy-4-*O*-methyl benzyl)chroman-4-one) [117].

#### 3.2.6. *Stuhlmannia moavi*

The previously discovered HIF bonducellin has been isolated and purified from the root EtOAc-soluble partition of *Stuhlmannia moavi*, and SLH CC (eluent: CH_2_Cl_2_–MeOH 1:1) and recrystallization techniques were exploited [118].

### 3.3. Homoisoflavonoids Isolated from Miscellaneous Plant Families

#### 3.3.1. Portulacaceae Family

*Portulaca oleracea*, belonging to Portulacaceae family, is extensively distributed all over the world. In folk medicine, it has been acknowledged to be a natural antispasmodic, diuretic, antiseptic, antiscorbutic, analgesic, etc. agent [119,120,121,122]. Besides all the identified phytoconstituents from this species, several HIFs were isolated and described (Table S3).

An EtOAc fraction of the aerial parts of *P. oleracea* was subjected to CC on silica gel (mobile phase: CHCl_3_–MeOH) which led to the isolation of four novel HIFs: portulacanone A (syn. 2′-hydroxy- 5,7-dimethoxy-3-benzylchroman-4-one), portulacanone B (syn. 2′-hydroxy-5,6,7-trimethoxy-3-benzyl-chroman-4-one), portulacanone C (syn. 5,2′-dihydroxy-6,7-dimethoxy-3-benzyl-chroman-4-one), and portulacanone D (syn. 5,2′-dihydroxy-7-methoxy-3-benzylidene-chroman-4-one) [123].

HPLC of a hydro-methanolic (85%) soluble partition of *P. oleracea* yielded the known compounds portulacanone A and E (isocratic eluent: CHCl_3_–MeOH 95:5), and portulacanone C and B (gradient eluent: H_2_O–MeCN 43:57 to 47:53) [124]. Portulacanone A and D have also been isolated by means of CC on silica gel (gradient mobile phase: CHCl_3_–MeOH 100:0 to 0:100) and HPLC (isocratic solvent system H_2_O–MeOH 75:25) from a hydro-methanolic extract [125].

Portulacanones E and F have been isolated for the first time from the same plant extract as above by employing mobile phase systems of H_2_O–MeOH with ratios of 70:30 and 55:45 in HPLC, respectively [126]. Moreover, the CH_2_Cl_2_-soluble partition of *P. oleracea* has been subjected to RP-CC (H_2_O–MeOH 50:50 to 0:100 as gradient mobile phase), CC on silica gel (eluent: CHCl_3_–MeOH 100:0 to 0:100), and finally HPLC (mobile phase: H_2_O–MeOH 75:25) to isolate three known HIFs, namely, 5,7-dimethoxy-3-(2’-hydroxybenzyl)-4-chromanone, 5-hydroxy-6,7-dimethoxy-3-(2’-hydroxybenzyl)-4-chromanone, and (*E*)-5-hydroxy-7-methoxy-3-(2′-hydroxybenzyl)-4-chromanone [127,128].

#### 3.3.2. Cucurbitaceae Family

Only one species from the Cucurbitaceae family, *Cucumis bisexualis,* has been established for its HIF contents (Table S3). The fruits were extracted with EtOAc and four novel HIFs, including 3-(4′-hydroxybenzylidene)-8-(3″,3″-dimethylfuran-2″-one)-6,7-dimethoxy-chroman-4-one, 3-(3′-methoxy-4′-hydroxybenzylidene)-8-(3″,3″-dimethyl-furan-2″-one)-7-methoxy-chroman-4-one, 3-(benzo-dioxol-1′-ylmethylene)-8-(3″,3″-dimethyl-furan-2″-one)-6-hydroxy-chroman-4-one, and 3-(benzo-dioxol-1′-ylmethylene)-8-(3″,3″-dimethyl-furan-2″-one)-6-hydroxy-5,7-dimethoxychroman-4-on, along with eight derivatives, which were new in this species, were isolated and characterized. CC on silica gel by applying a gradient mobile phase of PET–EtOAc (6:1 to 2:1) and SLH CC (mobile phase: H_2_O–MeOH 1:9) were utilized as chromatographic procedures [129].

#### 3.3.3. Polygonaceae Family

As shown in Table S3, two species of *Polygonum* have been reported to contain HIFs. The aerial part of *P. senegalense* has been used in the extraction process; after solvent–solvent partitioning, the Me_2_CO fraction was chromatographed on silica gel CC employing mobile phase systems of PET–Bz–CHCl_3_, MeOH, and PET–CHCl_3_; the novel homoisoflavanone 5,7-dihydroxy-3-(hydroxy-phenyl-methyl)-6-methoxy-chroman-4-one (syn. polygohomoisoflavanone) was accordingly isolated [130].

*P. ferrugineum* has also been studied for its phytochemical contents. The leaf CH_2_Cl_2_-soluble partition was initially subjected to vacuum liquid chromatography (VLC) using the mobile phases CHCl_3_–EtOAc and Me_2_CO–MeOH, then CC on silica gel (mobile phase: NHEX–EtOAc; EtOAc–MeOH; MeOH), finally to PTLC (eluent: CHCl_3_–EtOAc–HCO_2_H 90:10:1). As a consequence, the novel homoisoflavanone derivative 5,7-dihydroxy-6-methoxy-3-(9-hydroxy-phenylmethyl)-chroman-4-one could be isolated [131].

### 3.4. Gan Luo Xin, a Chinese Herbal Medicine

“Gan Luo Xin” is described in Chinese folk medicine as a traditional herbal preparation to remedy hepatitis B. It is composed of 20 various plant species such as *Panax ginseng*, *Astragalus membranaceus*, *Polygonatum sibiricum*, and *Crataegus pinnatifida*. Its 1-butanol-soluble extract has been subjected to HPLC separation by applying an isocratic mobile phase system H_2_O–MeOH (60:40), affording four HIFs. Among them, two derivatives named (±)-5,7-dihydroxy-8-methyl-3-(2′,4′-dihydroxybenzyl)chroman-4-one and (±)-5,7-dihydroxy-6,8-dimethyl-3-(2′,4′-dihydroxybenzyl)chroman-4-one were characterized as novel secondary metabolites. It may be implied that those HIFs are correlated to *Polygonatum sibiricum* content in this folk prescription (Table S3) [132].

## 4. Structural Identification of Homoisoflavonoids

The structural characterization of HIFs has relied on various spectroscopic techniques, mainly nuclear magnetic resonance (NMR), mass spectrometry (MS), spectrophotometric ultraviolet (UV), and infrared (IR). Both proton (^1^H) and carbon-13 (^13^C) NMR spectra in one-dimensional (1D) or 2D experiments such as ^1^H–^1^H correlation spectroscopy (COSY), nuclear Overhauser effect spectroscopy (NOESY), heteronuclear multiple bond correlation (HMBC), and heteronuclear single quantum coherence (HSQC) have been applied to the structure elucidation of these compounds. Since homoisoflavonones possess a five-proton spin system on carbons 2, 3, and 9, they are able to be characterized according to their protons’ chemical shifts in those positions. The homoisoflavans are usually identified by the analysis of the seven-proton spin system spread over carbons 2, 3, 4, and 9; ring A of HIFs has two or three substituents, while ring B can be recognized for its oxygen substituent at position 4′ (Figure 1). The determination of physical properties, such as melting point, plays a role. Moreover, in several studies, circular dichroism (CD) and optical rotatory power ([α]D) were utilized to ascertain the absolute stereochemistry of the HIF structures.

## 5. Conclusions and Perspectives

HIFs are a small class of flavonoids with privileged biological activities. According to the literature, these bioactive phenolics are mainly present in Asparagaceae, Fabaceae, Portulacaceae, Cucurbitaceae, and Polygonaceae families. The tuberous root of *Ophiopogon japonicus* (Asparagaceae) is the richest source of HIFs, specifically its EtOAc-soluble fraction. The EtOAc partition of the *Polygonatum odoratum* rhizome is known for its diverse HIF derivatives. From the plants belonging to the Fabaceae family, the heartwood parts of *Heamatoxylon campechianum* and *Caesalpinia* spp., particularly *C. sappan*, are considered as a striking natural source of these compounds. To date, over 300 HIFs have been reported in the literature [1]. The preponderance of information documented herein was to overview the chemotaxonomic standpoint of HIFs and separation techniques used for their isolation. They do not only display a broad structural diversity but are also known for a variety of biological properties and some of these compounds stand out for their unique pharmacological properties. More phytochemical and pharmacological studies will be required to fully exploit the potential of these interesting compound class substances.

## Data Availability

Not applicable.

## Supplementary Materials

The following are available online at https://www.mdpi.com/1422-0067/22/5/2735/s1, Table S1: Isolated homoisoflavonoid derivatives from Asparagaceae family, Table S2: Homoisoflavonoid derivatives isolated from Fabaceae family, Table S3: Homoisoflavonoids isolated from plant families except Asparagaceae and Fabaceae.
