# Supplementary material for: A Comprehensive Review on Chemotaxonomic and Phytochemical Aspects of Homoisoflavonoids, as Rare Flavonoid Derivatives
Source: Int J Mol Sci. 2021 Mar 8;22(5):2735. doi: 10.3390/ijms22052735 (PMC7962952; doi:10.3390/ijms22052735)
Supplement: Supplementary file 1 [file ijms-22-02735-s001.zip › Table S1-2021.02.12.pdf]

Table S1. Isolated homoisoflavonoid derivatives from Asparagaceae family

Plant species	Subjected soluble fraction/plant part	Method of isolation/purification	Name	Reference
Agave sisalana	EtOAc/L	HPLC [NHEX–EtOAc 7:1; NHEX–EtOAc–Me_2CO 7:1:0.5]	7-*O*-methyleucomol	[73]
			3′-deoxysappanon	
			(±)-3,9-dihydroeucomin	
		HPLC [NHEX–EtOAc 3.5:1; NHEX–EtOAc–Me_2CO 7:1:1]	dihydro-bonducellin	
			7-hydroxy-3-(4-hydroxybenzyl)chroman	
		HPLC [NHEX–EtOAc 3:1]	5,7-dihydroxy-3-(4′-hydroxy-benzyl)-4-chromanone	
			5,7-dihydroxy-3-(3′-hydroxy-4′-methoxybenzyl)-4-chromanone	
Agave tequilana	EtOAc/Fr	CC [NHEX–EtOAc 9:1 to 1:9], FC [NHEX–EtOAc 8:2]	5,7-dihydroxy-3-(4-methoxybenzyl)-chroman-4-one	[74]
	Me_2CO/Fr		7-hydroxy-3-(4-hydroxybenzyl)-chroman-4-one	
			4′-demethyl-3,9-dihydro-punctatin	
Albuca fastigiata	CH_2Cl_2/bulb	CC [CH_2Cl_2–MeOH 99:1]	3,5-dihydroxy-7-methoxy-3-(4′-hydroxy-3′-methoxybenzyl)-4-chromanone	[79]
Bellevalia eigii	MeOH–MeCN/bulb	HPLC [MeOH–H_2O (0.1% HCO_2H) 70:30 to 90:10]	7-*O*-methyl-3,9-dihydropunctatin	[53]
			7-*O*-methylpunctatin	
		HPLC [MeOH–H_2O (0.1% HCO_2H) 50:50 to 60:40]	8-*O*-demethyl-7-*O*-methyl-3,9-dihydropunctatin	
			7-*O*-methyl-3′-hydroxy-3,9-dihydropunctatin	
			5,7-dihydroxy-3-(3′-hydroxy-4′-methoxybenzyl)-4-chromanone	
			5,6-dihydroxy-3-(4-hydroxybenzyl)-7-methoxy-4-chromanone	
			6-hydroxy-7-*O*-methyl-3,9-dihydropunctatin	
			7,4′-di-*O*-methyl-3′-hydroxy-3,9-dihydropunctatin	
			demethyleucomin	
			punctatin	
			7-*O*-methyl-3′-hydroxypunctatin	

			5-hydroxy-7,8-dimethoxychroman-4-one	
			7-*O*-methyl-8-demethoxy-3-hydroxy-3,9-dihydropunctatin	
			isomuscomosin	
			4′,5,7-trihydroxyhomoisoflavanone	
		HPLC [MeOH–H_2O (0.1% HCO_2H) 50:50 to 100:0]	3′,4′,5,7-tetrahydroxyhomoisoflavanone	
Bellevalia flexuosa	$CHCl_3$/bulb	HPLC [MeCN–H_2O (0.1% HCO_2H) 40:60 to 50:50]	4′,5,7-trihydroxyhomoisoflavanone	[54]
			isomuscomosin	
			3-(4-hydroxybenzyl)-5-hydroxy-6,7,8-trimethoxychroman-4-one	
			7-*O*-methyl-8-demethoxy-3-hydroxy-3,9-dihydropunctatin	
			7,4′-*O*-dimethyl-8-demethoxy-3,3′-dihydroxy-3,9-dihydropunctatin	
			3′-hydroxy-3,9-dihydroeucomin	
		HPLC [MeCN–H_2O (0.1% HCO_2H) 40:60 to 60:40]	3,9-dihydropunctatin	
			7-*O*-methyl-8-demethoxy-3′-hydroxy-3,9-dihydropunctatin	
			8-*O*-demethyl-7-*O*-methyl-3,9-dihydropunctatin	
			7-*O*-methyl-3′-hydroxypunctatin	
			7-*O*-methylpunctatin	
			7,4′-di-*O*-methyl-3′-hydroxy-3,9-dihydropunctatin	
		HPLC [MeCN–H_2O (0.1% HCO_2H) 40:60 to 50:50] and [MeOH–H_2O (0.1% HCO_2H) 60:40 to 70:30]	7-*O*-methyl-3,9-dihydropunctatin	
		HPLC [MeCN–H_2O (0.1% HCO_2H) 30:70 to 40:60]	6-hydroxy-8-demethoxy-4′-*O*-methyl-3,9-dihydropunctatin	
		HPLC [MeCN–H_2O (0.1% HCO_2H) 28:72]	6-hydroxy-7-*O*-methyl-3,9-dihydropunctatin	
			7-*O*-methyl-3-hyroxy-3,9-dihydropunctatin	
		HPLC [MeOH–H_2O (0.1% HCO_2H) 50:50 to 60:40]	5-hydroxy-7,8-dimethoxychroman-4-one	
Bellevalia saviczii	CH_2Cl_2/R	MPLC [MeOH–H_2O 20:80 to 100:0]	dracol	[55]
Bessera elegans	MeOH/bulb	CC [$CHCl_3$–MeOH–H_2O 100:10:1], RP-CC [MeOH–H_2O 2:1]	(3*R*)-5,7-dihydroxy-6-methyl-3-(3′-hydroxy-4′-methoxybenzyl)chroman-4-one	[80]
			(3*R*)-5,7,3′-trihydroxy-4′-methoxy-6-methylspiro[2*H*-1-benzopyran-7′-bicyclo[4.2.0]octa[1,3,5]-trien]-4-one	
Chlorophytum inornatum	NHEX/R	SLH, RP-PTLC [MeOH–H_2O 85:15]	3-(4′-methoxybenzyl)-7,8-methylenedioxy-chroman-4-one	[81]
Disporopsis aspera	EtOAc/rhizome	PTLC [NHEX–EtOAc 4:6]	3-(2′,4′-dihydroxybenzyl)-5,7-dihydroxy-chroman-4-one (syn. disporopsin)	[82]

		CC [NHEX–EtOAc 1:0, 9:1, 8:2, 7:3, 6:4, 1:1], PTLC [NHEX–EtOAc 6:4]	3-(4′-hydroxy-benzyl)-5,7-dihydroxy-6-methyl-8-methoxy-chroman-4-one	
		CC [NHEX–EtOAc 1:0, 9:1, 8:2, 7:3, 6:4, 1:1], PTLC [NHEX–EtOAc 7:3]	3-(4′-hydroxy-benzyl)-5,7-dihydroxy-6-methylchroman-4-one	
			3-(4′-hydroxy-benzyl)-5,7-dihydroxy-6,8-dimethyl-chroman-4-one	
Dracaena cambodiana	EtOAc/St	CC [$CHCl_3$–MeOH 50:1 to 30:1], SLH [EtOH]	cambodianol	[56]
Dracaena cinnabari	$CHCl_3$/resin	CC [NHEX–EtOAc], HPLC [MeOH–H_2O (0.2% HOAC)]	7-hydroxy-3-(3-hydroxy-4-methoxybenzyl)chroman	[57]
		CC [NHEX–EtOAc]	7-hydroxy-3-(4-hydroxybenzyl)-8-methoxychroman	
			3-(4-hydroxybenzyl)-7,8-methylenedioxychroman	
			7-hydroxy-3-(4-hydroxybenzyl)chroman	
Dracaena cochinchinensis	EtOAc/resin (Chinese dragon's blood)	SLH [MeOH], HPLC [MeCN–H_2O 35:65]	(7*R*,12b*R*)-7,10-dihydroxy-4,11-dimethoxydracaenone	[59]
			10-hydroxy-11-methoxydracaenone	
			(7*S*,12b*S*)-11-hydroxy-1,10-dimethoxydracaenone	
			(7*S*,12b*S*)-10,11-dihydroxy-1-methoxydracaenone	
		SLH [MeOH], HPLC [MeOH–H_2O 58:42]	(3*S*)-3,7,4′-trihydroxy-5-methoxyhomoisoflavanonol	
			(3*R*)-7,4′-dihydroxy-6-methoxyhomoisoflavane	
		CC [PE–EtOAc 5:1 to 0:1]	7,4′-dihydroxyhomoisoflavanone	
			loureiriol	
		HPLC [MeOH–H_2O 30:70]	7,4′-dihydroxy-5-methoxyhomoisoflavanone	
			5,4′-dihydroxy-7- methoxyhomoisoflavone	
		RP-CC [MeOH–H_2O 4:6 to 1:0], CC [$CHCl_3$–MeOH 20:1 to 5:1], RP-CC [MeOH–H_2O 1:1 to 1:0]	dracaeconolide A	[60]
			(3*R*)-6,4′-dihydroxy-8-methoxyhomoisoflavan	
			dracaeconolide B	
			(3*R*)-7,4′-dihydroxy-8-methoxyhomoisoflavane	
			(3*R*)-7-hydroxy-3-(4-hydroxybenzyl)chromane	
			(3*R*)-7,4′-dihydroxy-5-methoxy-homoisoflavane	
	MeOH/resin	SLH [MeOH], HPLC [MeCN–H_2O 44:56 to 47:53, MeOH–H_2O 65:35 to 67:33]	biflavocochin A	[61]
			biflavocochin G	
			biflavocochin E	
			biflavocochin B	
		HPLC [MeCN–H_2O 50:50]	biflavocochin D	
			biflavocochin F	
	EtOAc/St	CC, SLH	10,11-dihydroxydracaenone C	[62]

			7,4′-dihydrohomoisoflavanone	
			7,4′-homoisoflavane	
Dracaena draco	Me_2CO/resin	CC [CH_2Cl_2–MeOH 99:1, 95:5, 90:10, 85:5, 80:20], PTLC [NHEX–$CHCl_3$]	7-hydroxy-3-(4-hydroxybenzyl)chromane	[64]
			5,7-dihydroxy-6-methoxy-3-(4-hydroxy-benzyl)-chroman-4-one	
		CC [CH_2Cl_2–MeOH 99:1, 95:5, 90:10, 85:5, 80:20], FC [NHEX–EtOAc 1:0, 9:1, 8:2, 7:3, 6:4, 1:1], PTLC [NHEX–EtOAc 8:2; NHEX–$CHCl_3$ 3:1]	7-hydroxy-3-(4-hydroxybenzyl)-8-methylchromane	
			5,7-dihydroxy-6,8-dimethyl-3-(4-hydroxy-benzyl)-chromane-4-one	
			7-hydroxy-3-(4-hydroxybenzyl)-8-methoxylchromane	
			7-hydroxy-3-(4-hydroxybenzyl)-5-methoxychromane	
		CC [CH_2Cl_2–MeOH 99:1, 95:5, 90:10, 85:5, 80:20], PTLC [$CHCl_3$–EtOAc 9:1]	7-hydroxy-3-(4-hydroxybenzyl)-chroman-4-one	
			5,7-dihydroxy-3-(4-hydroxy-benzyl)-chroman-4-one	
		CC [CH_2Cl_2–MeOH 99:1, 95:5, 90:10, 85:5, 80:20], CC [NHEX–EtOAc]	5,7-dihydroxy-6-methyl-3-(4-hydroxy-benzyl)-chromane-4-one	
			5,8-dihydroxy-7-methoxy-3-(4-hydroxy-benzyl)-chroman-4-one	
	EtOH/L	SLH [NHEX–CH_2Cl_2–MeOH 2:1:1] and PTLC [NHEX–EtOAc 85:15]	(3*R*)-2,3-dihydro-3,5-dihydroxy-7-methoxy-3-[(4-methoxyphenyl)-methyl]-8-methyl-4H-[1]benzopyran-4-one (syn. dracol)	[63]
		SLH [NHEX–CH_2Cl_2–MeOH 2:1:1] and PTLC [CH_2Cl_2–Me_2CO 15:1, toluene–*i*-PrOH 10:1]	5,7-dihydroxy-3-(4-hydroxybenzyl)chromone	
Dracaena loureiri	n.d/St	CC, PTLC	(7*S*, 12b*R*)-10-hydroxy-11-methoxy-dracaenone	[65]
			(7*R*, 12b*R*)-7,10-dihydroxy-11-methoxy-dracaenone	
			(3*S*)-7,4-dihydroxy-3-(4-hydroxybenzyl)chromane	
			(3*R*)-eucomol	
Drimia delagoensis	CH_2Cl_2/bulb	CC [EtOAc–$CHCl_3$ 2:10]	5,7-dihydroxy-(4-hydroxy-3-methoxybenzyl)chroman-4-one	[83]
Drimiopsis barteri	MeOH–$CHCl_3$/L, bulb	CC [$CHCl_3$–PE 7:3], PTLC [$CHCl_3$–PE–MeOH 7:2:0.5]	5,6,7-trihydroxy-3-(4-hydroxybenzyl)chroman-4-one	[84]
Eucomis autumnalis	*n*-BuOH/bulb	CC [NHEX–CH_2Cl_2 50:50 to 0:100], CC [CH_2Cl_2–EtOAc 50:50], CC [CH_2Cl_2–MeOH 50:50]	3R-(4′-hydroxybenzyl)-6,8-dihydroxy-5,7-dimethoxy-4-chromanone	[67]
Eucomis comosa	EtOAc/bulb	CC [EtOAc (0 to 100%)–NHEX]	7-hydroxy-5-methoxy-3-(4′-hydroxybenzyl)-4-chromanone	[66]
			5,7-dihydroxy-8-methoxy-3-(4′-hydroxybenzyl)-4-chromanone (syn. 3,9-dihydropunctatin)	
			punctatin	
			(*Z*)-eucomin	
			(*E*)-eucomin	

Eucomis montana	CH_2Cl_2/bulb	CC [CH_2Cl_2]	3,9-dihydroeucomin	[68]
			7-*O*-methyleucomol	
		CC [EtOAc–CH_2Cl_2 1:9]	eucomol	
		CC [EtOAc–CH_2Cl_2 3:17]	5,6-dimethoxy-7-hydroxy-3-(4′-hydroxybenzyl)-4-chromanone	
	EtOAc/bulb	CC [CH_2Cl_2]	4′-demethyl-5-*O*-methyl-3,9-dihydroeucomin	
			eucomin	
		CC [EtOAc–CH_2Cl_2 1:9]	8-*O*-demethyl-7-*O*-methyl-3,9-dihydropunctatin	
		CC [EtOAc–CH_2Cl_2 1:4]	7-*O*-methyl-3,9-dihydropunctatin	
			3′,5,7-trihydroxy-4′-methoxyspiro[2H-1-benzopyran-3(4H),7′-bicyclo[4.2.0]octa-1,3,5-trien]-4-one	
	MeOH/bulb	CC [EtOAc–CH_2Cl_2 2:3]	4′-demethyl-3,9-dihydroeucomin	
			4′-*O*-demethyleucomol	
Eucomis pallidiflora subsp. *pole-evansii*	CH_2Cl_2/bulb	CC [CH_2Cl_2–MeOH 98:2]	8-methoxy-5,6,7-trihydroxy-3-(4′-hydroxybenzylidene)-4-chromanone	[66]
Eucomis schijffii	CH_2Cl_2/bulb	CC [CH_2Cl_2]	scillascillin	[66]
Eucomis vandermerwei	CH_2Cl_2/bulb	CC [CH_2Cl_2]	(*E*)-5,7-dihydroxy-3-(4′-methoxybenzylidene)-4-chromanone	[69]
			(*R*)-5,7-dihydroxy-3-(4′-methoxybenzyl)-4-chromanone	
	MeOH/bulb	CC [CH_2Cl_2–MeOH 98:2]	3,5,7-trihydroxy-3-(4′-methoxybenzyl)-4-chromanone	
		CC [CH_2Cl_2–MeOH 95:5]	(*R*)-5,6-dimethoxy-7-hydroxy-3-(4′-hydroxybenzyl)-4-chromanone	
Eucomis zambesiaca	MeOH/bulb	CC [CH_2Cl_2–MeOH 98:2]	(*R*)-5,7-dihydroxy-8-methoxy-3-(4′-hydroxybenzyl)-4-chromanone	[69]
			(*R*)-5,7-dihydroxy-6-methoxy-3-(4′-hydroxybenzyl)-4-chromanone	
			(*R*)-5,7-dihydroxy-8-methoxy-3-(4′-methoxybenzyl)-4-chromanone	
		CC [CH_2Cl_2–MeOH 99:1]	(*E*)-5,7-dihydroxy-8-methoxy-3-(4′-methoxybenzylidene)-4-chromanone	
			(*E*)-5,7-dihydroxy-3-(4′-methoxybenzylidene)-4-chromanone	
Herreria montevidensis	$CHCl_3$/R	SLH [MeOH] and HPLC [MeOH–H_2O 5:5]	(3*R*)-7-hydroxy-8-methoxy-3-(4-hydroxybenzyl)chroman	[85]
			(3*R*)-7-hydroxy-8-methoxy-3-(4-hydroxybenzyl)chroman	
		HPLC [MeOH–H_2O 5:5]	(3*R*)-7-methoxy-3-(4-hydroxybenzyl)chroman	
			(3*R*)-7-hydroxy-5-methoxy-6-methyl-3-(4-hydroxybenzyl)chroman	

			(3*R*)-7-hydroxy-5-methoxy-6-methyl-3-(4-hydroxybenzyl)chroman	
			(3*R*)-7-methoxy-3-(4-hydroxybenzyl)chroman	
		HPLC [MeOH–H_2O 7:3]	(3*R*)-5,7-dimethoxy-6-methyl-3-(4-hydroxybenzyl)chroman	
			(3*R*)-5,7-dimethoxy-6-methyl-3-(4-hydroxybenzyl)chroman	
	MeOH/R	HPLC [MeOH–H_2O 7:3]	7-hydroxy-8-methoxy-3-(4-hydroxybenzyl)-3-chromen	
			7-hydroxy-8-methoxy-3-(4-hydroxybenzyl)-3-chromen	
Ledebouria graminifolia	CH_2Cl_2–MeOH/bulb	SLH, PTLC [$CHCl_3$–MeOH 95:5]	5-hydroxy-7-methoxy-3-(4′-hydroxybenzyl)-4-chromanone	[70]
			5-hydroxy-6,7-dimethoxy-3-(4′-hydroxybenzyl)-4-chromanone	
			5,7,8-trimethoxy-3-(4′-hydroxybenzyl)-4-chromanone	
			5-hydroxy-3′,4′,7-trimethoxyspiro{2H-1-benzopyran-7′-bicyclo[4.2.0]octa[1,3,5]-trien}-4-one	
			5,7-dihydroxy-3′,4′-dimethoxyspiro{2H-1-benzopyran-7′-bicyclo[4.2.0]octa[1,3,5]-trien}-4-one	
Ledebouria ovatifolia	CH_2Cl_2/bulb	CC [NHEX–CH_2Cl_2], CC [CH_2Cl_2–MeOH]	2-hydroxy-7-*O*-methylscillascillin	[71]
			(*R*)-5,7-dihydroxy-3-(4′-hydroxybenzyl)chroman-4-one	
			(*R*)-5,7-dihydroxy-3-(4′-methoxybenzyl)chroman-4-one	
			(*R*)-7-hydroxy-3-(4′-hydroxybenzyl)-5-methoxychroman-4-one	
	EtOAc/bulb	n.d	(*R*)-3-(3′,4′-dihydroxybenzyl)-7-hydroxy-5-methoxychroman-4-one	
			(*E*)-3-(3′,4′-dihydroxybenzylidene)-7-hydroxy-5-methoxychroman-4-one	
			(*E*)-3-(3′,4′-dihydroxybenzylidene)-5,7-dihydroxychroman-4-one (syn. ovatifolionone)	
Ledebouria socialis	CH_2Cl_2/bulb	CC [NHEX–CH_2Cl_2], CC [CH_2Cl_2–MeOH], PTLC [EtOAc–CH_2Cl_2 10:90]	(*R*)-2′,5-dihydroxy-3′,4′,7-trimethoxyspiro{2H-1-benzopyran-3-(4H)-9-bicyclo[4.2.0]octa[1,3,5]triene}-4-one (syn. socialinone)	
Liriope platyphylla	hydro-ethanolic/R	CC [CH_2Cl_2–MeOH 98:2], SLH [CH_2Cl_2–EtOAc–MeOH 1:1:6], HPLC [MeOH–H_2O 65:35]	(−)-liriopein A	[86]
		CC [CH_2Cl_2–MeOH 96.5:3.5], RP-CC [MeOH–H_2O 75:25]	(−)-liriopein B	
		SLH [CH_2Cl_2–EtOAc–MeOH 1:1:6], RP-CC [MeOH–H_2O 65:35]	(3*R*)-3-(4′-hydroxybenzyl)-5,7-dihydroxylchroman-4-one	
			3-(4′-hydroxybenzylidene)-5,7-dihydroxychroman-4-one	

		SLH [CH_2Cl_2–EtOAc–MeOH 1:1:6], HPLC [MeOH–H_2O 75:25]	(3*R*)-3-(4′-hydroxybenzyl)-5,7-dihydroxy-6-methyl-chroman-4-one	
		RP-CC [MeOH–H_2O 60:40], HPLC [MeOH–H_2O 50:50]	(3*R*)-3-(2′,4′-dihydroxybenzyl)-5,7-dihydroxychroman-4-one	
			(3*R*)-3-(2′,4′-dihydroxybenzyl)-5,7-dihydroxy-6-methyl-chroman-4-one	
		RP-CC [MeOH–H_2O 75:25]	(3*R*)-3-(4′-hydroxybenzyl)-3,5-dihydroxy-7-methoxy-6-methylchroman-4-one	
	EtOAc/AP	CC [CH_2Cl_2–MeOH 20:1 to 15:1], HPLC [MeCN–H_2O 45:55]	3-(2',4'-dihydroxybenzyl)-5,7-dihydroxy-6-methylchroman-4-one	[87]
		HPLC [MeOH–H_2O 60:40]	3-(2',4'-dihydroxy-benzyl)-5,7-dihydroxy-6-methylchroman-4-one	
Massonia bifolia	EtOH/bulb	CC [CH_2Cl_2–EtOAc], SLH [CH_2Cl_2–MeOH 1:1]	(*R*)-(4′-hydroxy)-5-hydroxy-7-methoxy-4-chromanone	[72]
			(*R*)-(4′-hydroxy)-5,7-dihydroxy-4-chromanone	
			(*E*)-3-benzylidene-(3′,4′-dihydroxy)-5-hydroxy-7-methoxy-4-chromanone	
			(*R*)-(3′-hydroxy-4′-methoxy)-5,7-dihydroxy-4-chromanone	
			(*E*)-3-(3′,4′-dihydroxybenzylidene)-5-hydroxy-7-methoxy-4-chromanone	
Massonia pustulata	CH_2Cl_2/bulb	CC [CH_2Cl_2–EtOAc], SLH [CH_2Cl_2–MeOH 1:1]	(*R*)-5-hydroxy-3-(4-hydroxybenzyl)-7-methoxy-4-chromanone	[73]
			5-hydroxy-3-(4-hydroxybenzyl)-7-methoxy-4-chromone	
Ophiopogon japonicus	ether/subterranean	CC [Bz], recrys.	methylophiopogonone A	[22]
			methylophiopogonanone A	
			5-hydroxy-7, 8-dimethoxy-6-methyl-3-(3', 4'-dihydroxybenzyl)chroman-4-one	
		CC [NHEX–Me_2CO 8:2], recrys.	ophiopogonanone A	
			ophiopogonone A	
		HPLC [MeCN–H_2O 1:1]	desmethylisoophiopogonone B	
			5,7,2′-trihydroxy-6-methyl-3-(3′, 4′-methylenedioxybenzyl)chromone	
			5,7,2′-trihydroxy-8-methyl-3-(3′, 4′-methylenedioxybenzyl)chromone	
	EtOH–EtOAc/tuber	recrys. [$CHCl_3$–MeOH]	ophiopogonanone C	[21]
		SLH [MeOH–H_2O 4:1]	ophiopogonanone D	
			ophiopogonone C	

		PTLC [$CHCl_3$–EtOAc 20:1]	ophiopogonanone E	
			ophiopogonanone F	
			5,7,2′-trihydroxy-6-methyl-3-(3′,4′-methylenedioxybenzyl) chromone	
			2′-hydroxymethylophiopogonone A	
		CC [NHEX–$CHCl_3$]	6-aldehydoisoophiopogonone A	
	$CHCl_3$/tuber	RP-CC [Me_2CO–H_2O 3:2]	homoisopogon A	[24]
			homoisopogon C	
		RP-CC [Me_2CO–H_2O 1:1]	homoisopogon B	
			homoisopogon D	
	EtOAc/R	HPLC [MeOH–H_2O 7:3]	ophiopogonone D	[25]
			ophiopogonanone G	
	EtOAc/tuberous R	FC [NHEX–$CHCl_3$ 7:3, $CHCl_3$]	5,7-dihydroxy-8-methoxy-6-methyl-3-(2′-hydroxy-4′-methoxybenzyl)chroman-4-one	[26]
		FC [$CHCl_3$–MeOH 97:3], PTLC [$CHCl_3$–MeOH 95:5]	7-hydroxy-5,8-dimethoxy-6-methyl-3-(2′-hydroxy-4′-methoxybenzyl)chroman-4-one	
		FC [$CHCl_3$–MeOH 95:5]	5,7-dihydroxy-6,8-dimethyl-3-(4′-hydroxy-3′-methoxybenzyl)chroman-4-one	
		FC [$CHCl_3$–MeOH 95:5], PTLC [$CHCl_3$–MeOH 93:7], SLH [EtOH]	2,5,7-trihydroxy-6,8-dimethyl-3-(3′,4′-methylenedioxybenzyl)-chroman-4-one	
			2,5,7-trihydroxy-6,8-dimethyl-3-(4′-methoxybenzyl)chroman-4-one	
		CC [$CHCl_3$], PTLC [$CHCl_3$–MeOH 93:7, NHEX–EtOAc 1:1]	5,7-trihydroxy-6,8-dimethyl-3-(2′-hydroxy-3′,4′-methylenedioxybenzyl)chromone	
			methylophiopogonone A	
			ophiopogonanone A	
		CC [$CHCl_3$], SLH [EtOH]	ophiopogonanone B	
			methylophiopogonanone A	
		CC [NHEX–$CHCl_3$ 7:3], PTLC [$CHCl_3$]	6-formyl-isoophiopoganone A	
		CC [$CHCl_3$], PTLC [$CHCl_3$–MeOH 93:7], RP-CC [MeOH–H_2O 8:2]	5-hydroxy-7,8-dimethoxy-6-methyl-3-(3′,4′-dihydroxybenzyl)chroman-4-one	
		RP-CC [MeOH–H_2O 8:2], PTLC [NHEX–EtOAc 1:1]	5,7-dihydroxy-6,8-dimethyl-3-(4′-hydroxy-3′,8′-dimethoxybenzyl)chroman-4-one	
	EtOAc/R	HPLC [MeCN–H_2O (0.1% HCO_2H) 75:25]	ophiopogonanone G	[10]
		HPLC [MeOH–H_2O (0.1% HCO_2H) 80:20]	ophiopogoside A	

			ophiopogoside B	
	hydro-ethanolic (80%)/tuberous R	HPLC [MeOH–H_2O 60:40 to 70:30]	ophiopogonone E	[27]
			ophiopogonanone H	
			5,7,4′-trihydroxy-3′-methoxy-6,8-dimethyl homoisoflavanone	
			5,7-dihydroxy-4′-methoxy-6-methyl homoisoflavanone	
			methylophiopogonanone B	
			5,7,2′-trihydroxy-4′-methoxy-6,8-dimethyl homoisoflavanone	
			ophiopogonanone A	
			methylophiopogonanone A	
		HPLC [MeOH–H_2O 65:35]	5,7,2′-trihydroxy-3′,4′-methylenedioxy-6,8-dimethyl homoisoflavone	
			methylophiopogonone A	
		SLH [MeOH]	ophiopogonanone E	
			5,7,2′-trihydroxy-3′,4′-methylenedioxy-8-methyl homoisoflavone	
		SLH [MeOH], HPLC [MeOH–H_2O 55:45 to 60:40]	7,2′-dihydroxy-5,8,4′-trimethoxy-6-methyl homoisoflavanone	
			5,7,4′-trihydroxyhomoisoflavone	
			5,7,4′-trihydroxy-6-methyl homoisoflavanone	
	MeOH/R	HSCCC [NHEX–EtOAc–MeOH–MeCN–H_2O 1.8:1.0:1.0:1.2:1.0]	methylophiopogonanone A	[28]
			6-aldehydo-isoophiopogonone A	
			6-formyl-isoophiopogonanone A	
	Et_2O/tuber	SLH [MeOH], SLH [EtOH]	methylophiopogonanone A [syn. 5,7-dihydroxy-6,8-dimethyl-3-(3,4-methylenedioxybenzyl)-chroman-4-one]	[29]
			methylophiopogonanone B [syn. 5,7-dihydroxy-6,8-dimethyl-3-(4-methoxybenzyl)-chroman-4-one]	
		SLH [MeOH], CC [$CHCl_3$–Me_2CO 95:5]	methylophiopogonone A [syn. 5,7-dihydroxy-6,8-dimethyl-3-(3,4-methylenedioxybenzyl)-chromone]	
			methylophiopogonone B [syn. 5,7-dihydroxy-6,8-dimethyl-3-(4-methyoxybenzyl)-chromone]	
			isoophiopogonone A	
		SLH [MeOH], CC [$CHCl_3$–Me_2CO 95:5], SLH [EtOH]	ophiopogonone A	
			ophiopogonone B	
	Hydroethanolic (70%)/rhizome	CC [$CHCl_3$–MeOH 100:0 to 85:15], SLH [$CHCl_3$–MeOH 1:1], CC [$CHCl_3$–MeOH 99:1]	ophiopogonanone E	[30]

		CC [$CHCl_3$–MeOH 100:0 to 85:15], SLH [$CHCl_3$–MeOH 1:1], HPLC [MeCN–H_2O 35:65]	desmethylisoophiopogonone B (syn. 5,7-dihydroxy-3-(4′-hydroxybenzyl)-8-methylchromoe)	
			5,7-dihydroxy-6-methyl-3-(4′-hydroxy-benzyl)-chroman-4-one	
			5,7-dihydroxy −6-methyl-3-(4′-hydroxybenzyl)chromone	
			3-(2,4-dihydroxybenzyl)-5-hydroxyl- 7,8-dimethoxy-6-methylchroman-4-one	
		CC [$CHCl_3$–MeOH 100:0 to 85:15], SLH [$CHCl_3$–MeOH 1:1]	5,7-dihydroxy-3-(4′-hydroxybenzyl) chromone	
			4′-*O*-demethylophiopogonanone E	
			ophiopogonone D	
			5,7- dihydroxy-6-methyl-3-(2′,4′-dihydroxybenzyl)chroman-4-one	
	EtOAc/R	CC [PE–EtOAc 50:1, 20:1, 10:1, 5:1, 2:1], HSCCC [NHEX–EtOAc–MeOH–MeCN–H_2O 3:2:3.5:1:0.5; 3:2:2.5:1:1.5]	6-aldehydo-isoophiopogonanone A	[31]
			6-aldehydo-isoophiopogonanone B	
			methylophiopogonanone A	
			methylophiopogonanone B	
Ornithogalum dubium Houtt.	EtOH/bulb	CC [CH_2Cl_2], SLH [MeOH–CH_2Cl_2]	(3*S*)-5-hydroxy-7-methoxy-3-(4-methoxybenzyl)-4-chromanone	[88]
			(3*R*)-5-hydroxy-7-methoxy-3-(4-methoxybenzyl)-4-chromanone	
			(3*S*)-5-hydroxy-7-methoxy-3-(4-hydroxybenzyl)-4-chromanone	
			(3*R*)-3,5-dihydroxy-7-methoxy-3-(4′-hydroxybenzyl)-4-chromanone	
			(3*R*)-3-hydroxy-7-methoxy-3-(4-hydroxybenzyl)-4-chromanone 5-*O*-β-D-glucopyranoside	
			(3*R*)-3-hydroxy-7-methoxy-3-(4-hydroxybenzyl)-5-*O*-β-D-glucopyranosyl-(1→6)-β-D-glucopyranoside-4-chromanone	
Polygonatum cyrtonema	EtOAc/rhizome	CC [PE–EtOAc 100:1 to 2:1], CC [NHEX–Me_2CO 4:1], SLH [$CHCl_3$–MeOH 1:1], PTLC [NHEX–Me_2CO 3:1]	(3*R*)-5,7-dihydroxy-8-methyl-3-(2′-hydroxy-4′-methoxybenzyl)-chroman-4-one	[42]
	petroleum ester/rhizome	CC, HPLC	(3*R*)-5,7-dihydroxy-6-methyl-3-(2′-hydroxy-4′-methoxybenzyl)-chroman-4-one (syn. polygonatone H)	[43]
		CC [petroleum ester–Me_2CO 1:0 to 0:1], SLH [$CHCl_3$–MeOH 1:1], HPLC [MeCN–H_2O 25:75 to 60:40]	5,7-dihydroxy-6,8-dimethyl-3-(4′-hydroxybenzyl)-chroman-4-one	

			5,7-dihydroxy-6,8-dimethyl-3-(2′-methoxy-4′-hydroxybenzyl)-chroman-4-one	
		CC [petroleum ester–Me_2CO 1:0 to 0:1], HPLC [MeCN–H_2O 25:75 to 60:40]	5,7-dihydroxy-6-methyl-3-(4′-hydroxybenzyl)-chroman-4-one	
			5,7-dihydroxy-8-methyl-3-(4′-hydroxybenzyl)-chroman-4-one	
			5,7-dihydroxy-6-methyl-3-(4′-methoxybenzyl)-chroman-4-one	
		CC [petroleum ester–Me_2CO 80:20 to 0:100], SLH [$CHCl_3$–MeOH 1:1], HPLC [MeCN–H_2O 25:75 to 60:40]	5,7-dihydroxy-6,8-dimethyl-3-(4′-methoxybenzyl)-chroman-4-one	
			5,7-dihydroxy-3-(4′-methoxybenzyl)-chroman-4-one	
			disporopsin	
	EtOAc/rhizome	CC, HPLC	5,7-dihydroxy-3-(4′-hydroxybenzyl)-chroman-4-one	
			5,7-dihydroxy-6-methyl-3-(2′,4′-dihydroxybenzyl)-chroman-4-one	
			5,7-dihydroxy-3-(2′-hydroxy-4′-methoxybenzyl)-chroman-4-one	
			5-dihydroxy-7-methoxy-6,8-dimethyl-3-(2′-hydroxy-4′-methoxybenzyl)-chroman-4-one	
			polygonatone D	
			5,7-dihydroxy-3-(4′-hydroxybenzylidene)-chroman-4-one	
Polygonatum odoratum	PE/rhizome	CC	(3*R*)-5,7-dihydroxy-8-methoxy-3-(4-methoxybenzyl)-6-methylchrom-an-4-one (syn. odoratumone A)	[32]
			(3*R*)-5,7,8-trihydroxy-3-(4-hydroxybenzyl)-6-methylchroman-4-one (syn. odoratumone B)	
	EtOAc/rhizome	CC [PE–Me_2CO 98:2, 95:5, 90:10, 80:20, 50:50], CC [PE–EtOAc 50:1, 25:1, 10:1, 5:1], SLH [$CHCl_3$–MeOH 4:1]	3-(4′-methoxy-benzyl)-5,7-dihydroxy-6-methyl-8-methoxy-chroman-4-one (syn. methylodoratumanone A)	[33]
			3-(4′-methoxy-benzyl)-5,7-dihydroxy-6,8-dimethyl-chroman-4-one	
		CC [PE–Me_2CO 98:2, 95:5, 90:10, 80:20, 50:50], SLH [$CHCl_3$–MeOH 4:1]	3-(4′-hydroxy-benzyl)-5,7-dihydroxy-6,8-dimethyl-chroman-4-one	
			3-(4′-hydroxy-benzyl)-5,7-dihydroxy-6-methyl-8-methoxy-chroman-4-one	
			3-(4′-hydroxy-benzyl)-5,7-dihydroxy-6-methyl-chroman-4-one	

		CC [PE–Me_2CO 98:2, 95:5, 90:10, 80:20, 50:50], SLH	3-(4′-hydroxy-benzyl)-5,7-dihydroxy-6-methyl-8-methoxy-chroman-4-one	
			3-(4′-hydroxyl-benzyl)-5,7-dihydroxy-6-methyl-chroman-4-one	
	EtOAc/R	HPLC [MeCN–H_2O 40:60 (0.15% triethylamine, 0.18% formic acid)]	2,3-dihydro-3-[(15-hydroxyphenyl)methyl]-5,7-dihydroxy-6-methyl-8-methoxy-4H-1-benzopyran-4-one	[34]
		CC [$CHCl_3$–MeOH–H_2O 100:0:0 to 50:50:5], CC [PE–EtOAc 95:5 to 50:50], CC [PE–EtOAc 9:1 to 7:3], SLH [$CHCl_3$–MeOH 1:1], recrys.	5,7-dihydroxy-6,8-dimethyl-3(*R*)-(3′-hydroxy-4′-methoxybenzyl)-chroman-4-one	[35]
		CC [$CHCl_3$–MeOH–H_2O 100:0:0 to 50:50:5], CC [PE–EtOAc 95:5 to 50:50], CC [PE–EtOAc 9:1 to 7:3], SLH [$CHCl_3$–MeOH 1:1], CC [$CHCl_3$–MeOH 95:5]	(±)-5,7-dihydroxy-6,8-dimethyl-3-(2′-hydroxy-4′-methoxybenzyl)-chroman-4-one	
			5,7-dihydroxy-6,8-dimethyl-3-(4′-hydroxybenzyl)chroman-4-one	
		CC [$CHCl_3$–MeOH–H_2O 100:0:0 to 50:50:5], CC [PE–EtOAc 95:5 to 50:50], CC [PE–EtOAc 9:1 to 7:3], SLH [$CHCl_3$–MeOH 1:1], SLH [MeOH]	(*E*)-5,7-dihydroxy-6,8-dimethyl-3-(4′-hydroxybenzylidene)chroman-4-one	
		CC [$CHCl_3$–MeOH–H_2O 100:0:0 to 50:50:5], CC [EtOH–H_2O 0:100, 30:70, 50:50, 70:30, 95:5], SLH [MeOH–H_2O 8:2], SLH [MeOH]	4′-demethylleucomin 7-*O*-*β*-D-glucopyranoside	
		CC [$CHCl_3$–MeOH–H_2O 100:0:0 to 50:50:5], CC [PE–EtOAc 95:5 to 50:50], CC [PE–Me_2CO 8.5:1.5]	5,7-dihydroxy-6-methyl-8-methoxy-3-(4′-methoxybenzyl)chroman-4-one	
		CC [$CHCl_3$–MeOH–H_2O 100:0:0 to 50:50:5], CC [PE–EtOAc 95:5 to 50:50], CC [PE–EtOAc 8:2], SLH [$CHCl_3$–MeOH 1:1]	methylophiopogonanone B	
		CC [$CHCl_3$–MeOH–H_2O 100:0:0 to 50:50:5], CC [PE–EtOAc 95:5 to 50:50], PTLC [$CHCl_3$–MeOH 95:5]	5,7-dihydroxy-6-methyl-8-methoxy-3-(4′-hydroxybenzyl)chroman-4-one	
			ophiopogonanone E	
	$CHCl_3$/rhizome	SLH, PTLC	(*E*)-3-(3,4-dihydroxybenzylidene)-5,7-dihydroxy-6,8-dimethylchroman-4-one	[36]
			(*E*)-3-(3,4-dihydroxybenzylidene)-5,7-dihydroxy-8-methoxy-6-methylchroman-4-one	
	EtOH/rhizome	SLH and HPLC	(3*R*)-5,7-dihydroxyl-6-methyl-8-methoxyl-3-(4′-hydroxylbenzyl)-chroman-4-one	[37]
			(3*R*)-5,7-dihydroxyl-6,8-dimethyl-3-(4′-hydroxylbenzyl)-chroman-4-one	
			(3*R*)-5,7-dihydroxyl-6-methyl-3-(4′-hydroxylbenzyl)-chroman-4-one	

			polygonatone A	
			polygonatone B	
			polygonatone C	
	EtOAc/n.d	PTLC	5,7-dihydroxy-6,8-dimethyl-3-(4′-hydroxybenzyl)chroman-4-one	[38]
		CC [NHEX–EtOAc]	5,7-dihydroxy-6-methyl-8-methoxy-3-(4′-hydroxybenzyl)chroman-4-one	
	EtOAc/rhizome	HPLC [MeCN–H_2O (0.18% HCO_2H) 60:40]	5,7-dihydroxy-3-(4′-hydroxybenzyl)-6-methylchroman-4-one	[39]
			5,7-dihydroxy-3-(4′-hydroxybenzyl)-6-methyl-8-methoxychroman-4-one	
			5,7-dihydroxy-3-(4′-hydroxybenzyl)-6, 8-dimethylchroman-4-one	
	$CHCl_3$/rhizome	RP-CC [MeOH–H_2O 70:30 to 100:0], HPLC [MeOH–H_2O 60:40]	3-(4′-hydroxybenzyl)-5,7-dihydroxy-6-methyl-8-methoxychroman-4-one	[40]
		RP-CC [MeOH–H_2O 70:30 to 100:0], HPLC [MeOH–H_2O 65:35]	3-(4′-hydroxybenzyl)-5,7-dihydroxy-6,8-dimethylchroman-4-one	
		RP-CC [MeOH–H_2O 70:30 to 100:0], HPLC [MeOH–H_2O 70:30]	3-(4′-methoxybenzyl)-5,7-dihydroxy-6-methyl-8-methoxychroman-4-one	
	hydroethanolic (60%)/rhizome	HSCCC [PE–EtOAc–MeOH–H_2O 2:3:3:2], SLH [MeOH–MeCN 1:1]	(3*R*)-5,7-dihydroxy-8-methyl-3-(2′,4′-dihydroxybenzyl)-chroman-4-one	[41]
			(3*R*)-5,7-dihydroxy-8-methyl-3-(4′-hydroxybenzyl)-chroman-4-one	
			(3*R*)-5,7-dihydroxy-3-(2′-hydroxy-4′-methoxybenzyl)-chroman-4-one	
			(3*R*)-5,7-dihydroxy-3-(4′-hydroxybenzyl)-chroman-4-one	
			(3*R*)-5,7-dihydroxy-6-methoxy-8-methyl-3-(2′,4′-dihydroxybenzyl)-chroman-4-one	
			(3*R*)-5,7-dihydroxy-8-methoxy-3-(2′-hydroxy-4′-methoxybenzyl)-chroman-4-one	
			(3*R*)-5,7-dihydroxy-6-methyl-3-(4′-hydroxybenzyl)-chroman-4-one	
			(3*R*)-5,7-dihydroxy-6-methyl-8-methoxy-3-(4′-hydroxybenzyl)-chroman-4-one	
			(3*R*)-5,7-dihydroxy-6,8-dimethyl-3-(4′-hydroxybenzyl)-chroman-4-one	

			(3*R*)-5,7-dihydroxy-6-methyl-8-methoxy-3-(4′-methoxybenzyl)-chroman-4-one	
		HSCCC [PE–EtOAc–MeOH–H_2O 2:3:3:2], recrys.	(3*R*)-5,7-dihydroxy-3-(2′,4′-dihydroxybenzyl)-chroman-4-one	
Polygonatum verticillatum	$CHCl_3$–EtOAc/rhizome	CC [Bz–EtOAc 10:0 to 0:10], RP-CC [MeOH–H_2O 70:30]	5,7-dihydroxy-3-(4-methoxybenzyl)-8-methyl chroman-4-one	[44]
		CC [Bz–EtOAc 10:0 to 0:10], CC [NHEX–EtOAc 80:20]	5,7-dihydroxy-3-(2-hydroxy-4-methoxybenzyl)-8-methylchroman-4-one	
		CC [Bz–EtOAc 10:0 to 0:10], CC [NHEX–EtOAc 85:15]	5,7-dihydroxy-3-(2-hydroxy-4-methoxybenzyl)-chroman-4-one	
Pseudoprospero firmifolium	CH_2Cl_2/bulb	CC [CH_2Cl_2–EtOAc]	3,5-dihydroxy-7,8-dimethoxy-3-(3′,4′-dimethoxybenzyl)-4-chromanone	[89]
			3,5-dihydroxy-7-methoxy-3-(3′,4′-dimethoxybenzyl)-4-chromanone	
			3,5-dihydroxy-7,8-dimethoxy-3-(3′-hydroxy-4′-methoxybenzyl)-4-chromanone	
			3,5,6-trihydroxy-7-methoxy-3-(3′-hydroxy-4′-methoxybenzyl)-4-chromanone	
			3,5,7-trihydroxy-3-(3′-hydroxy-4′methoxybenzyl)-4-chromanone	
Resnova humifusa	CH_2Cl_2/bulb	CC [EtOAc–CH_2Cl_2 3:2]	4′-demethyl-3,9-dihydroeucomin	[68]
			5,6-dimethoxy-7-hydroxy-3-(4′-hydroxybenzyl)-4-chromanone	
			8-*O*-demethyl-7-*O*-methyl-3,9-dihydropunctatin	
			4′-*O*-demethyleucomol	
			eucomol	
			3,9-dihydroeucomin	
		CC [EtOAc–CH_2Cl_2 1:4]	5,7-dihydroxy-3-(3′-hydroxy-4′-methoxybenzyl)-4-chromanone	
			7-*O*-methyl-3,9-dihydropunctatin	
			3′,5,7-trihydroxy-4′-methoxyspiro[2H-1-benzopyran-3(4H),7′-bicyclo[4.2.0]octa-1,3,5-trien]-4-one	
	MeOH/bulb	CC [CH_2Cl_2–MeOH 98:2]	(*R*)-5,7-dihydroxy-8-methoxy-3-(4′-hydroxybenzyl)-4-chromanone	[69]
			(*R*)-7,8-dimethoxy-5-hydroxy-3-(4′-hydroxybenzyl)-4-chromanone	

			(*R*)-5,8-dihydroxy-7-methoxy-3-(4′-hydroxybenzyl)-4-chromanone	
		CC [CH_2Cl_2–MeOH 99:1]	(*R*)-7-hydroxy-5-methoxy-3-(4′-hydroxybenzyl)-4-chromanone	
Rhodocodon aff. intermedius	CH_2Cl_2/bulb	FC [NHEX, NHEX–CH_2Cl_2, MeOH], CC [MeOH–NHEX–CH_2Cl_2 1:4:5]	(*E*)-5,6,7-trihydroxy-3-(3′-hydroxy-4′-methoxybenzylidene)-4-chromanone	[75]
			(*E*)-5,7-dihydroxy-3-(3′-hydroxy-4′-methoxybenzylidene)-4-chromanone	
			(3*S*)-5,6-dihydroxy-7-methoxy-3-(3′-hydroxy-4′-methoxybenzyl)-4-chromanone	
Rhodocodon campanulatus	EtOH/bulb	SLH [CH_2Cl_2–MeOH 1:1], CC	(3*S*)-5,7-dihydroxy-3S-(3′-hydroxy-4′-methoxybenzyl)-4-chromanone	[75]
		n.d	2,5,7-dihydroxy-3-(3-hydroxy-4-methoxybenzyl) chroman-4-one	[76]
Rhodocodon cryptopodus	EtOH/bulb	CC	3*S*-5,7-dihydroxy-(3′-hydroxy-4′-methoxybenzyl)-4-chromanone	[77]
Rhodocodon rotundus	EtOH/bulb	FC	3*S*-5,7-dihydroxy-(4′-hydroxy-3′-methoxybenzyl)-4-chromanone	[77]
Scilla dracomontana	ether/bulb	CC	5,7-dihydroxy-6-methoxy-3-(4-methoxybenzyl)chroman-4-one	[48]
			eucomol	
Scilla kraussii	ether/bulb	CC	5,7-dihydroxy-3-(3-hydroxy-4-methoxybenzyl)chroman-4-one	[48]
Scilla natalensis	NHEX/bulb	CC	5,7-dihydroxy-6-methoxy-3-(3-methoxybenzyl)chroman-4-one	[48]
			5,7-dihydroxy-6-methoxy-3-(4-hydroxybenzyl)chroman-4-one	
Scilla nervosa	NHEX, EtOAc/bulb	n.d	5,7-dimethoxy-3-(4-methoxybenzyl)chroman-4-one	[48]
			5-hydroxy-7-methoxy-3-(3-hydroxy-4-methoxy-benzyl)chroman-4-one	
			5,7-dimethoxy-3-(4-hydroxybenzyl)chroman-4-one	
	EtOAc/bulb	n.d	5-hydroxy-7-methoxy-3-(4-methoxybenzyl)chroman-4-one	
			5,7-dimethoxy-3-(4-hydroxy benzylidene)chroman-4-one	
Scilla nervosa subsp. *rigidifolia*	EtOAc/bulb	PTLC	3-(4′-methoxybenzyl)-6,7-dihydroxy-5-methoxychroman-4-one	[45]

			3-(3′-hydroxy-4′-methoxybenzyl)-5,7-dimethoxychroman-4-one	
			3-(4′-methoxybenzylidene)-5,7-dihydroxy-6-methoxychroman-4-one	
			3-(4′-hydroxybenzylidene)-5,7-dihydroxy-6-methoxychroman-4-one	
			3-(4′-hydroxybenzylidene)-5,7-dihydroxychroman-4-one	
		SLH [$CHCl_3$–MeOH 7:3], PTLC	3-(4′-methoxybenzyl)-6-hydroxy-5,7-dimethoxychroman-4-one	
			3-(4′-methoxybenzyl)-5,7-dimethoxychroman-4-one	
	MeOH–$CHCl_3$ (20:80)/bulb	CC [$CHCl_3$–EtOAc 4:1]	3-(4-hydroxybenzylidene)-5,7-dihydroxychroman-4-one	[46]
			3-(4-hydroxybenzylidene)-5,7-dihydroxy-6-methoxychroman-4-one	
		CC [NHEX–$CHCl_3$], PTLC [$CHCl_3$]	3-(4-hydroxy-3-methoxybenzyl)-5-hydroxy-6,7-dimethoxychroman-4-one	
			3-(4-methoxybenzyl)-8-hydroxy-5,7-dimethoxychroman-4-one	
			3-(3,4-dimethoxybenzyl)-5,7-dihydroxychroman-4-one	
			3-(4-hydroxybenzylidene)-5-hydroxy-7-methoxychroman-4-one	
			3-(4-methoxybenzylidene)-5,7-dihydroxy-6-methoxychroman-4-one	
		FC [NHEX–$CHCl_3$ 1:1]	3-(4-methoxybenzyl)-5,7-dimethoxychroman-4-one	
		PTLC	3-(4-hydroxy-3-methoxybenzyl)-5-hydroxy-7-methoxychroman-4-one	
		PTLC [$CHCl_3$–EtOAc 9:1]	3-(4-hydroxybenzyl)-5,6,7-trimethoxychroman-4-one	
			3-(3-hydroxy-4-methoxybenzyl)-5,7-dihydroxychroman-4-one	
		recrys. [CH_2Cl_2]	3-(4-hydroxybenzyl)-5,7-dihydroxy-6-methoxychroman-4-one	
		CC [$CHCl_3$–EtOAc 9:1], CC [NHEX–EtOAc]	3-(4-methoxybenzyl)-6-hydroxy-5,7-dimethoxychroman-4-one	
	NHEX/bulb	CC [$CHCl_3$–EtOAc, EtOAc–MeOH], PTLC [$CHCl_3$–MeOH 24:1]	3-(4′-methoxybenzyl)-5,6,7-trimethoxychroman-4-one	[47]
		SLH [$CHCl_3$–EtOAc 7:3]	3-(4′-methoxybenzyl)-5,7-dimethoxychroman-4-one	

		SLH, PTLC	3-(4′-methoxybenzyl)-7-hydroxy-5,6-dimethoxychroman-4-one	
			3-(4′-methoxybenzyl)-6-hydroxy-5,7-dimethoxychroman-4-one	
		PTLC [$CHCl_3$–MeOH 24:1]	3-(3′-hydroxy-4′-methoxybenzyl)-5,7-dihydroxy-6-methoxychroman-4-one	
			3-(3′-hydroxy-4′-methoxybenzyl)-5,7-dihydroxychroman-4-one	
		SLH [$CHCl_3$–MeOH 95:5], PTLC	3-(4′-hydroxybenzylidene)-5,7-dihydroxy-6-methoxychroman-4-one	
			3-(4′-hydroxybenzylidene)-5,7-dihydroxychroman-4-one	
Scilla persica HAUSSKN	$CHCl_3$/bulb	SLH [MeOH]	3-(3′,4′-dihydroxybenzyl)-8-hydroxy-5,7-dimethoxychroman-4-one (syn. scillapersicone)	[51]
	Et_2O/bulb	SLH [MeOH]	3-(3′,4′-dihydroxybenzylidene)-8-hydroxy-5,7-dimethoxychroman-4-one (syn. scillapersicene)	[52]
			3-(3′,4′-dihydroxybenzyl)-5,8-dihydroxy-7-methoxychroman-4-one	
			3,9-dihydro-autumnalin	
			3-(3′,4′-dihydroxybenzylidene)-5,8-dihydroxy-7-methoxychroman-4-one	
			scillapersicone	
		CC [$CHCl_3$–MeOH 10:0 to 0:4]	autumnalin	
Scilla scilloides	EtOAc/bulb	CC, SLH, HPLC	(3*S*)-3-(3,4-dihydroxybenzyl)-5-hydroxy-6,7-dimethoxychroman-4-one (syn. scillavone B)	[50]
			3,9-dihydroeucomnalin	
			3-(3,4-dihydroxybenzyl)-5,7-dihydroxy-6-methoxychroman-4-one	
			3-(4-hydroxybenzylidene)-5,7-dihydroxychroman-4-one	
			3-(3,4-dihydroxybenzylidene)-5,7-dihydroxy-6-methoxy-chroman-4-one	
			(3*R*)-5,7,2′-trihydroxy-3′,4′-dimethoxyspiro{2*H*-1-benzopyran-7′-bicyclo[4,2,0]octa[1,3,5]-trien}-4-one (syn. scillavone A)	
			5,7,3′-trihydroxy-4′- methoxyspiro{2*H*-1-benzopyran-7′-bicyclo[4,2,0]octa[1,3,5]-trien}-4-one	

			scillascillin	
			2-hydroxy-scillascillin	
		HPLC [$CHCl_3$–MeOH 50:1]	3-(3,4-dihydroxybenzyl)-5,7-dihydroxy-6-methoxychroman-4-one	[49]
			3-(4-hydroxybenzylidene)-5,7-dihydroxychroman-4-one	
			scillavone A	
		HPLC [NHEX–Me_2CO 2:1]	3-(3,4-dihydroxybenzylidene)-5,7-dihydroxy-6-methoxychroman-4-one	
			scillavone B	
		HPLC [MeOH–H_2O 50:50], HPLC [$CHCl_3$–MeOH 50:1]	5,7,3′-trihydroxy-4′-methoxyspiro{2*H*-1-benzopyran-7′-bicyclo[4,2,0]octa[1,3,5]-trien}-4-one	
			2-hydroxy-scillascillin	
			3,9-dihydroeucomnalin	
		HPLC [MeOH–H_2O 65:35], recrys.	scillascillin	
Urginea depressa	CH_2Cl_2/WP	HPLC [MeCN–H_2O 3.5:10]	urgineanin A	[90]
			urgineanin D	
		HPLC [MeCN–H_2O 4:10]	urgineanin F	
	NHEX/WP	HPLC [MeCN–H_2O 4:10]	urgineanin B	
		HPLC [MeCN–H_2O 3.5:10]	urgineanin C	
			urgineanin E	
Veltheimia viridifolia	PE/bulb	HPLC [NHEX–Et_20 8:2 to 0:10]	R(–)-3-(4-hydroxybenzyl)-5-hydroxy-6,7,8-trimethoxychroman-4-one	[91]
	Et_2O/bulb	HPLC [$CHCl_3$–MeOH 95:5]	muscomin	

AP: aerial part; Bz: benzene; CC: column chromatography; $CHCl_3$: chloroform; CH_2Cl_2: dichloromethane; Et_2O: diethyl ether; EtOAc: ethyl acetate; EtOH: ethanol; FC: flash chromatography; Fr: fruit; H_2O: water; HCO_2H: formic acid; HOAc: acetic acid; HPLC: high-performance liquid chromatography; HSCCC; high-speed counter-current chromatography; *i*-PrOH: isopropyl alcohol; L: leaf; MeOH: methanol; Me_2CO: acetone; MeCN: acetonitrile; MPLC: medium-pressure liquid chromatography; *n*-BuOH: butanol; n.d: not determined; NHEX: *n*-hexane; PE: petroleum ether; PTLC: preparative-thin layer chromatography; R: root; recrys.: recrystallization; RP-CC: reverse-phase column chromatography; RP-PTLC: reverse-phase preparative-thin layer chromatography; SLH: Sephadex® LH-20; St: stem; WP: whole part
