# Supplementary material for: A Comprehensive Review on Chemotaxonomic and Phytochemical Aspects of Homoisoflavonoids, as Rare Flavonoid Derivatives
Source: Int J Mol Sci. 2021 Mar 8;22(5):2735. doi: 10.3390/ijms22052735 (PMC7962952; doi:10.3390/ijms22052735)
Supplement: Supplementary file 1 [file ijms-22-02735-s001.zip › Table S2-2021.02.12.pdf]

Table S2. Homoisoflavonoid derivatives isolated from Fabaceae family

Plant species	Subjected soluble fraction/plant part	Method of isolation/purification	Name	Reference
Caesalpinia bahamensis	MeOH/R	FC [CH_2Cl_2–EtOAc], HPLC [H_2O (0.1% HCO_2H)–MeCN (0.1% HCO_2H)]	metasappanin [syn. 3-(2-hydroxy-4-methoxybenzyl)chromane-4,7-diol]	[92]
Caesalpinia bonduc	EtOH/bark	CC [NHEX–$CHCl_3$ 100:0 to 0:100; $CHCl_3$–MeOH 100:0 to 0:100], CC [$CHCl_3$–MeOH 85:15], PTLC [CH_2Cl_2–MeOH 75:25]	caesalpinianone	[5]
			6-*O*-methylcaesalpinianone	
Caesalpinia digyna	MeOH/R	CC [NHEX–EtOAc 65:35 to 60:40]	(*Z*)-7,8-dihydroxy-3-(4′-methoxybenzyl)chroman-4-one (syn. isointricatinol)	[93]
		CC [NHEX–EtOAc 75:25 to 65:35]	intricatinol	
		HPLC [MeOH–H_2O 60:40]	(*Z*)-7-hydroxy-8-methoxy-3-(4′-methoxybenzyl)chroman-4-one	
			8-methoxybonducellin	
		HPLC [MeOH–H_2O 75:25]	*Z*-eucomine	
			E-eucomine	
		HPLC [MeOH–H_2O 70:30]	isobonducellin	
			bonducellin	
		SLH	demethyleucomine	
Caesalpinia japonica	CH_2Cl_2/heartwood	PTLC [$CHCl_3$–EtOAc 9:1]	3'-deoxy-4-*O*-methylsappanol (syn. 3,7-dihydroxy-3-(4-hydroxybenzyl)-4-methoxychroman)	[94]
			4-*O*-methylepisappanol	
		CC	4-*O*-methylsappanol	
		PTLC [$CHCl_3$–EtOAc 7:1]	sappanol	
			episappanol	
			sappanone A	
		PTLC	sappanone B	
Caesalpinia latisiliqua	EtOAc/twig	HPLC [MeCN–H_2O 35:65]	(3*S*)-dihydrobonducellin 8-*O*-β-D-glucopyranoside	[95]
Caesalpinia millettii	Me_2CO/St	SLH [MeOH]	8-methoxyisobonducellin	[96]
			8-methoxybonducellin	
		CC [$CHCl_3$–MeOH 10:0 to 1:1], CC [NHEX–$CHCl_3$ 9:1]	eucomin	

		CC [NHEX–EtOAc 9:1]	bonducellin	
		SLH [MeOH], CC [NHEX–EtOAc 7:3]	intricatinol	
Caesalpinia sappan	EtOAc/heartwood	RP-CC [MeOH–H_2O 40:60]	sappanone A	[101]
			3′-deoxy-4-*O*-methylsappanol	
		RP-CC [MeCN–H_2O 60:40]	3-deoxysappanone B	
			sappanol	
			7,3′,4′-trihydroxy-3-benzyl-2*H*-chromene	
			episappanol	
		RP-CC [MeCN–H_2O 70:30]	sappanone B	
			4-(7-hydroxy-2,2-dimethyl-9*βH*-1,3,5-trioxa-cyclopenta[*α*]naphthalene-3-lymethyl)-benzene-1,2-diol	
		RP-CC [MeOH–H_2O 80:20]	4-*O*-methylepisappanol	
		SLH	4-*O*-methylsappanol	
		HPLC [MeOH–H_2O 30:70 (0.1% TFA)]	caesalpiniaphenol A	[102]
			3-deoxysappanone B	
			sappanone A	
		HPLC [MeOH–H_2O 40:60 (0.1% TFA)]	caesalpiniaphenol B	
		HPLC [MeOH–H_2O 35:65 (0.1% TFA)]	3′-deoxy-4-*O*-methylepisappanol	
			3′-deoxysappanone A	
		HSCCC [$CHCl_3$–MeOH–H_2O 4:3:2]	3′-deoxysappanol	[103]
			3-deoxysappanone B	
			4-*O*-methylsappanol	
			brazilin	
		CC [$CHCl_3$–MeOH 95:5], CC [$CHCl_3$–Me_2CO 95:5, 92:8, 88:12], SLH [MeOH–H_2O 70:30]	7,3′,4′-trihydroxy-3-benzyl-2H-chromene	[104]
			4-*O*-methylsappanol	
			4-*O*-methylepisappanol	
			3′-deoxy-4-*O*-methylsappanol	
		CC [$CHCl_3$–MeOH 95:5], CC [$CHCl_3$–Me_2CO 9:1, 85:15, 8:2, 7:3], CC [CH_2Cl_2–Me_2CO 92:8, 9:1, 85:15], SLH [MeOH–H_2O 60:40]	caesalpin J	
			protosappanin A	
		CC [$CHCl_3$–MeOH 100:0 to 70:30], CC [$CHCl_3$–MeOH 95:5 to 80:20], SLH [$CHCl_3$–	3′,4-di-*O*-methylepisappanol (syn. (3*R*,4*R*)-3,7-dihydroxy-3-(3′-methoxy-4′-hydroxybenzyl)-4-methoxychroman	
			caesalpiniaphenol F	

		MeOH 65:35], RP-CC [MeOH–H_2O 60:40], HPLC [MeOH–H_2O 50:50]		
	MeOH/heartwood	SLH [MeOH], PTLC	7-hydroxy-3-(4′-hydroxybenzylidene-chroman-4-one	[105]
			3,7-dihydroxy-3-(4′-hydroxybenzyl)-chroman-4-one	
			3,4,7-trihydroxy-3-(4′-hydroxybenzyl)-chroman	
			7-hydroxy-8-methoxy-3-(4′-methoxybenzylidene)-chroman-one	
			8-methoxybonduallin	
	n.d/heartwood	n.d	4-*O*-methylsappanol	[106]
			protosappanin A	
			brazilin	
			caeasalpin J	
	n.d	CPC [EtOAc–MeCN–H_2O 1:1:2]	sappanol	[107]
			brazilin	
Caesalpinia pulcherrima	$CHCl_3$–MeOH/AP	PTLC [NHEX–EtOAc 3:10]	[(3*E*)-3-(1,3-benzodioxol-5-ylmethylene)-2,3-dihydro-7-hydroxy-4*H*-1-benzopyran-4-one]	[108]
			[(3*E*)-2,3-dihydro-7-hydroxy-3-[(4-methoxyphenyl)methylene]-4*H*-1-benzopyran-4-one] (syn. bonducellin)	
		PTLC [NHEX–EtOAc 1:9]	[(3*E*)-3-(1,3-benzodioxol-5-ylmethylene)-2,3-dihydro-7-methoxy-4*H*-1-benzopyran-4-one]	
			[(3*E*)-2,3-dihydro-7-methoxy-3-[(4-methoxy-phenyl)methylene]-4*H*-1-benzopyran-4-one] (syn. 7-*O*-methyl bonducellin)	
			(3*E*)-2,3-dihydro-6,7-dimethoxy-3[(3-hydroxy-4-methoxyphenyl)methylene]-4*H*-1-benzopyran-4-one	
		PTLC [NHEX–EtOAc 4:10]	[(3*E*)-2,3-dihydro-3[(3,4-dihydroxyphenyl)methylene]-7-hydroxy-4*H*-1-benzopyran-4-one] (syn. sappanone A)	
			[(3*E*)-2,3-dihydro-7-hydroxy-3-[(3-hydroxy-4-methoxyphenyl)-methylene]-4*H*-1-benzopyran-4-one]	
		CC [NHEX–EtOAc 1.5:10]	[(3*E*)-2,3-dihydro-3-[(3,4-dimethoxyphenyl)methylene]-7-methoxy-4*H*-1-benzopyran-4-one]	
		PTLC [NHEX–EtOAc 3.5:10]	[(3*E*)-2,3-dihydro-3-[(2,4-dimethoxyphenyl)methylene]-7-hydroxy-4*H*-1-benzopyran-1-one] (syn. 2′-methoxybonducellin)	
	NHEX/WP	CC [NHEX–EtOAc 95:5]	(*E*)-7-methoxy-3-(4′-methoxybenzylidene)chroman-4-one	[109]
	Me_2CO/WP	CC [NHEX–EtOAc 75:25]	(*Z*)-7-hydroxy-3-(4′-methoxybenzylidene)chroman-4-one (syn. isobonducellin)	

		CC [NHEX–EtOAc 70:30]	(*E*)-7-hydroxy-3-(4′-methoxybenzylidene)chroman-4-one (syn. bonducellin)	
		CC [NHEX–EtOAc 60:40]	(*E*)-7-hydroxy-3-(2′,4′-dimethoxybenzylidene)chroman-4-one	
			(*E*)-7-hydroxy-3-(3′,4′,5′-trimethoxybenzylidene)chroman-4-one	
	Me_2CO/AP	CC [NHEX–EtOAc 60:40, 65:35]	isobonducellin	[110]
		CC [NHEX–EtOAc 60:40]	bonducellin	
		CC [NHEX–EtOAc 60:40]	isobonducellin	[110–112]
			bonducellin	
	$CHCl_3$/St	CC [$CHCl_3$–EtOAc], recrys. [$CHCl_3$, MeOH]	bonducellin	[113]
		CC [$CHCl_3$–EtOAc], PTLC [$CHCl_3$–EtOAc 3:1]	8-methoxybonducellin	
	n-BuOH/cork tissue	CC [NHEX–Me_2CO 5:1 to 1:1], HPLC [MeCN–H_2O 37:63], PTLC [$CHCl_3$–Me_2CO 12:1]	dihydrobonducellin	[114]
			2′-methoxydihydrobonducellin	
			2′-methoxybonducellin	
			isobonducellin	
		CC [NHEX–Me_2CO 5:1 to 1:1], recrys.	bonducellin	
Crotalaria pallida Ait	CH_2Cl_2/Se	SLH [CH_2Cl_2–MeOH 2:1], HPLC [MeOH–H_2O 58:42]	cropalliflavone A	[115]
			cropalliflavone B	
Heamatoxylon campechianum	EtOAc/St	HPLC [MeCN–H_2O 10:90 to 35:65]	hematoxylol	[14]
			hematoxylone	
			isohematoxylin	
			epihematoxylol	
		HPLC [MeCN–H_2O 10:90 to 30:70]	sappanone B	
			4-*O*-methylepihematoxylol	
			hematoxylene	
			4-*O*-methylsappanol	
			hematoxin	
		SLH [MeOH]	4-*O*-methylhematoxylol	
			epihematoxin	
			sappanol	
			3′-deoxy-*O*-methylsappanol	
		CC [$CHCl_3$–MeOH 40:1 to 6:1]	3′-deoxy-sappanone A	
	CH_2Cl_2/St	SLH [$CHCl_3$–MeOH 1:1]	sappanene	

		CC [Me_2CO–PE 3:1 to 1:1]	sappanone A	
		CC [$CHCl_3$–MeOH 100:1 to 50:1]	isoliquiritigenin	
			bonducellin	
		CC [Me_2CO–PE 6:1 to 3:1]	(*E*)-eucomin	
		CC [Me_2CO–PE 3:1 to 1:1]	butein	
			sappanchalcone	
		CC [$CHCl_3$–MeOH 100:1 to 40:1]	3′-deoxy-sappanchalcone	
	EtOAc/heartwood	RP-CC [MeOH–H_2O 10:90 to 50:50], HPLC [MeCN–H_2O 10:90 to 30:70]	hematoxylol B	[116]
			epihematoxylol B	
			caesalpin J	
		HPLC [MeCN–H_2O 10:90 to 35:65]	1′-*O*-methylhematoxylol B	
			1′-*O*-methylepihematoxylol B	
		CC [$CHCl_3$–MeOH 20:1 to 3:1], SLH [$CHCl_3$–MeOH 1:1]	hematoxylin	
	MeOH/heartwood	CC [CH_2Cl_2–Me_2CO]	hematoxylol A	[117]
			4-*O*-methylhematoxylol	
			hematoxin	
Hoffmanosseggia intricata	$CHCl_3$/R	CC [CH_2Cl_2–MeOH 95:5, 98:2], recrys.	intricatin (syn. 7,4′-dimethoxy-8-hydroxyhomoisoflavone)	[12]
			intricatinol (syn. 4′-methoxy-7,8-dihydroxyhomoisoflavone)	
Pterocarpus marsupium	Et_2O/heartwood	CC [Bz; Bz–EtOAc; Bz–EtOAc 7:3]	pteromarsupone (syn. 6-hydroxy-7-*O*-methyl-3-(3-hydroxy-4-*O*-methyl benzyl)chroman-4-one)	[118]
Stuhlmannia moavi	EtOAc/R	SLH [CH_2Cl_2–MeOH 1:1], recrys.	bonducellin	[119]

AP: aerial part; Bz: benzene; CC: column chromatography; $CHCl_3$: chloroform; CH_2Cl_2: dichloromethane; CPC: centrifugal partition chromatography; EtOAc: ethyl acetate; EtOH: ethanol; H_2O: water; HPLC: high-performance liquid chromatography; HSCCC; high-speed counter-current chromatography; L: leaf; MeOH: methanol; Me_2CO: acetone; MeCN: acetonitrile; *n*-BuOH: butanol; n.d: not determined; NHEX: *n*-hexane; PE: petroleum ether; PTLC: preparative-thin layer chromatography; R: root; recrys.: recrystallization; RP-CC: reverse-phase column chromatography; Se: seed; SLH: Sephadex® LH-20; St: stem; TFA: trifluoroacetic acid; WP: whole part
