# Supplementary material for: A Comprehensive Review on Chemotaxonomic and Phytochemical Aspects of Homoisoflavonoids, as Rare Flavonoid Derivatives
Source: Int J Mol Sci. 2021 Mar 8;22(5):2735. doi: 10.3390/ijms22052735 (PMC7962952; doi:10.3390/ijms22052735)
Supplement: Supplementary file 1 [file ijms-22-02735-s001.zip › Table S3-2021.02.12.pdf]

Table S3. Homoisoflavonoids isolated from plant families except Asparagaceae and Fabaceae

Plant species	Plant family	Subjected soluble fraction/plant part	Method of isolation or purification (the final steps)	Name	Reference
Cucumis bisexualis	Cucurbitaceae	EtOAc/Fr	CC [PE–EtOAc 6:1 to 2:1], SLH [MeOH–H_2O 9:1]	3-(4′-hydroxybenzylidene)-8-(3′′,3′′-dimethylfuran-2′′-one)-6,7-dimethoxy-chroman-4-one	[130]
				3-(3′-methoxy-4′-hydroxybenzylidene)-8-(3′′,3′′-dimethyl-furan-2′′-one)-7-methoxy-chroman-4-one	
				3-(benzo-dioxol-1′-ylmethylene)-8-(3′′,3′′-dimethyl-furan-2′′-one)-6-hydroxy-chroman-4-one	
				3-(benzo-dioxol-1′-ylmethylene)-8-(3′′,3′′-dimethyl-furan-2′′-one)-6-hydroxy-5,7-dimethoxychroman-4-one	
				7-*O*-methylpunctatin	
				7-*O*-methyl-3′-hydroxypunctatin	
				punctatin	
				(3*E*)-3-(1,3-benzodioxol-5-ylmethylene)-2,3-dihydro-7-hydroxy-4*H*-1-benzopyran-4-one	
				(3*E*)-3-(1,3-benzodioxol-5-ylmethylene)-2,3-dihydro-7-methoxy-4*H*-1-benzopyran-4-one	
				isointricatinol	
				3-(4′-methoxybenzylidene)-5,7-dihydroxy-6-methoxychroman-4-one	
				8-methoxybonducellin	
Polygonum senegalense	Polygonaceae	Me_2CO/AP	CC [PE–Bz–$CHCl_3$; MeOH], CC [PE–$CHCl_3$]	5,7-dihydroxy-3-(hydroxy-phenyl-methyl)-6-methoxy-chroman-4-one (syn. polygohomoisoflavanone)	[131]
Polygonum ferrugineum		CH_2Cl_2/L	VLC [$CHCl_3$–EtOAc; Me_2CO–MeOH], CC [NHEX–EtOAc; EtOAc–MeOH; MeOH], PTLC [$CHCl_3$–EtOAc–HCO_2H 90:10:1]	5,7-dihydroxy-6-methoxy-3-(9-hydroxy-phenylmethyl)-chroman-4-one	[132]
Portulaca oleracea	Portulacaceae	hydro-methanolic (85%)/n.d	HPLC [$CHCl_3$–MeOH 95:5]	portulacanone E	[125]
				portulacanone A	
			HPLC [MeCN–H_2O 57:43 to 53:47]	portulacanone C	

				portulacanone B	
			HPLC [MeOH–H_2O 30:70]	portulacanone E	[127]
				portulacanone A	
			HPLC [MeCN–H_2O 45:55]	portulacanone B	
				portulacanone C	
				portulacanone F	
			SLH [NHEX–$CHCl_3$–MeCN 5:44:1], HPLC [MeOH–H_2O 27:73]	portulacanone D	
			n.d	2,2′-dihydroxy-4′,6′-dimethoxychalcone	
			CC [$CHCl_3$–MeOH 100:0 to 0:100], HPLC [MeOH–H_2O 25:75]	portulacanone A	[126]
				portulacanone D	
		CH_2Cl_2/n.d	RP-CC [MeOH–H_2O 50:50 to 100:0], CC [$CHCl_3$–MeOH 100:0 to 0:100], HPLC [MeOH–H_2O 25:75]	5,7-dimethoxy-3-(2'-hydroxybenzyl)-4-chromanone	[128]
				5-hydroxy-6,7-dimethoxy-3-(2'-hydroxybenzyl)-4-chromanone	
				(*E*)-5-hydroxy-7-methoxy-3-(2′-hydroxybenzyl)-4-chromanone	[128,129]
		EtOAc/AP	CC [$CHCl_3$–MeOH 10:0, 9.5:0.5, 9:1, 8.5:1.5, 8:2, 7:3, 0:10; 40:1 to 20:1]	portulacanone A (syn. 2′-hydroxy- 5,7-dimethoxy-3-benzylchroman-4-one)	[124]
				portulacanone C (syn. 5,2′-dihydroxy-6,7-dimethoxy-3-benzyl-chroman-4-one)	
			CC [$CHCl_3$–MeOH 10:0, 9.5:0.5, 9:1, 8.5:1.5, 8:2, 7:3, 0:10], CC [$CHCl_3$–MeOH 30:1 to 10:1]	portulacanone B (syn. 2′-hydroxy-5,6,7-trimethoxy-3-benzyl-chroman-4-one)	
			CC [$CHCl_3$–MeOH 10:0, 9.5:0.5, 9:1, 8.5:1.5, 8:2, 7:3, 0:10], CC [$CHCl_3$–MeOH 10:1 to 8:1]	portulacanone D (syn. 5,2′-dihydroxy-7-methoxy-3-benzylidene-chroman-4-one)	
"Gan Luo Xin" Chinese herbal medicine	combining over 20 species	*n*-BuOH/n.d	HPLC [MeOH–H_2O 60:40]	(±)-5,7-dihydroxy-8-methyl-3-(2′,4′-dihydroxybenzyl)chroman-4-one	[133]
				(±)-5,7-dihydroxy-6,8-dimethyl-3-(2′,4′-dihydroxybenzyl)chroman-4-one	
				5,7-dihydroxy-6-methyl-3-(2′,4′-dihydroxybenzyl)chroman-4-one	
				disporopsin	

AP: aerial part; Bz: benzene; CC: column chromatography; $CHCl_3$: chloroform; CH_2Cl_2: dichloromethane; EtOAc: ethyl acetate; Fr: fruit; H_2O: water; HCO_2H: formic acid; HPLC: high-performance liquid chromatography; L: leaf; MeOH: methanol; Me_2CO: acetone; MeCN: acetonitrile; *n*-BuOH: butanol; n.d: not determined; NHEX: *n*-hexane; PE: petroleum ether; PTLC: preparative-thin layer chromatography; recrys.: recrystallization; RP-CC: reverse-phase column chromatography; SLH: Sephadex® LH-20
